# Atomic Electronic Structure Calculations with Hermite Interpolating Polynomials

**DOI:** 10.1021/acs.jpca.3c00729

**Published:** 2023-04-27

**Authors:** Susi Lehtola

**Affiliations:** † Molecular Sciences Software Institute, Blacksburg, Virginia 24061, United States; ‡ Department of Chemistry, University of Helsinki, P.O. Box 55, FI-00014 Helsinki, Finland

## Abstract

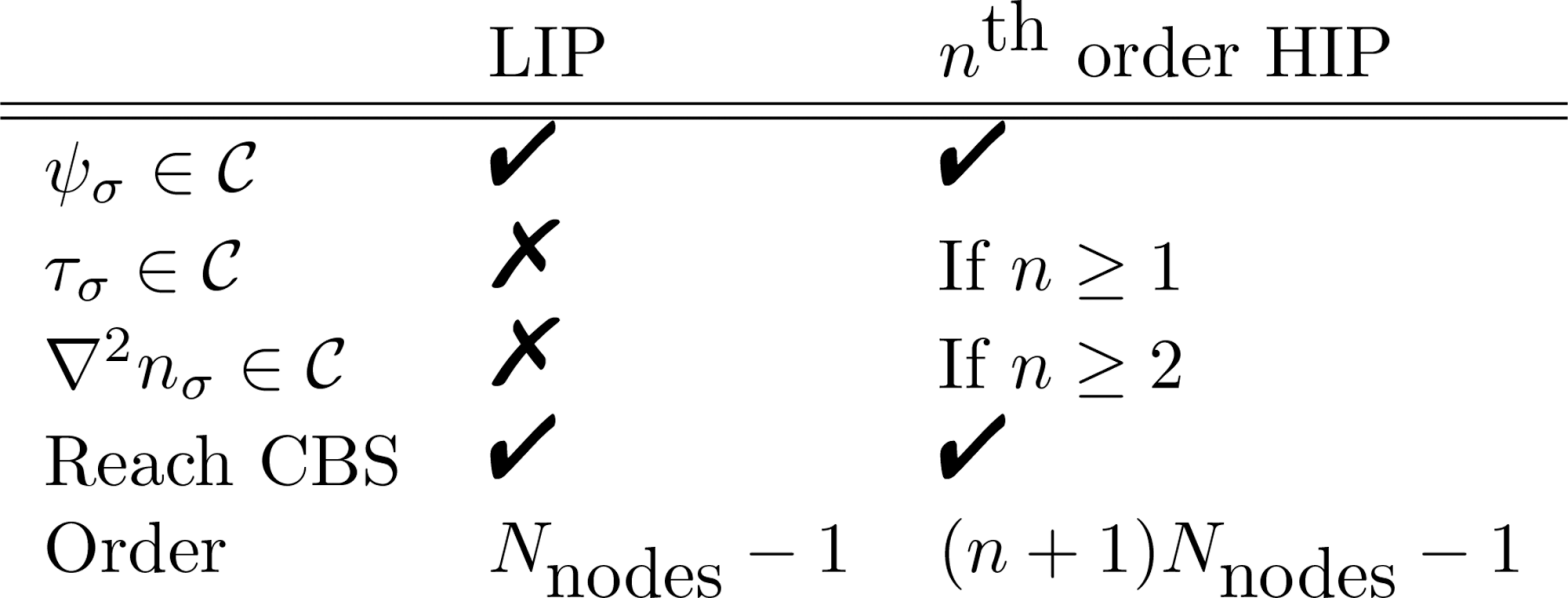

We have recently described the implementation of atomic electronic structure calculations within the finite element method with numerical radial basis functions of the form χ_μ_(*r*) = *r*^–1^*B*_μ_(*r*), where high-order Lagrange interpolating polynomials (LIPs) were used as the shape functions *B*_μ_(*r*). In this work, we discuss how χ_μ_(*r*) can be evaluated in a stable manner at small *r* and also revisit the choice of the shape functions *B*_μ_(*r*). Three kinds of shape functions are considered: in addition to the  continuous LIPs, we consider the analytical implementation of first-order Hermite interpolating polynomials (HIPs) that are  continuous, as well as numerical implementations of *n*-th order ( continuous) HIPs that are expressed in terms of an underlying high-order LIP basis. Furnished with the new implementation, we demonstrate that the first-order HIPs are reliable even with large numbers of nodes and that they also work with nonuniform element grids, affording even better results in atomic electronic structure calculations than LIPs with the same total number of basis functions. We demonstrate that discontinuities can be observed in the spin-σ local kinetic energy τ_σ_ in small LIP basis sets, while HIP basis sets do not suffer from such issues; however, either set can be used to reach the complete basis set limit with smooth τ_σ_. Moreover, we discuss the implications of HIPs on calculations with meta-GGA functionals with a number of recent meta-GGA functionals, and we find most Minnesota functionals to be ill-behaved. We also examine the potential usefulness of the explicit control over the derivative in HIPs for forming numerical atomic orbital basis sets, but we find that confining potentials are still likely a better option.

## 1 Introduction

In order to perform electronic structure calculations, the problem needs to be discretized to fit a computer. The first step in electronic structure theory is to determine the single particle states, usually known as molecular orbitals, which are almost invariably expanded in terms of analytic basis sets of a predefined form, as in the linear combination of atomic orbitals (LCAO) approach, for example. However, the goodness of the obtained solutions depends critically on the properties of the basis set used for the expansion: if the basis set is a poor fit to the problem, the results are not good either.

Even though the bound solutions of the hydrogenic problem, *Ĥ* = −∇^2^/2 – *Z*/*r*, naively sound like a good starting point for finding a polyatomic solution, such a basis is in fact a terrible starting point for electronic structure problems, as the set of bound hydrogenic solutions must be supplemented by the unbounded continuum states in order to form a complete basis set. It has been known for a very long time that the contribution from the continuum can be significant—comparable in magnitude to that of the bound solutions—in many cases;^1,2^ this problem has been recently discussed for solutions of the hydrogenic ground state problem for charge *Z*′ in the basis of the bound solutions for *Z* ≠ *Z*′ by Forestell and Marsiglio.^3^ For related reasons, the orbitals obtained from the one-electron part of the molecular Hamiltonian *Ĥ* = −∇^2^/2 – ∑_*A*_*Z*_*A*_/*r*_*A*_ are likewise a terrible guess for solving the self-consistent field (SCF) equations occurring in both Hartree–Fock and Kohn–Sham^4^ theory.^5^

Instead of the hydrogenic basis, most atomic-orbital calculations employ basis sets of simpler analytic form, such as Gaussian type orbitals and Slater type orbitals, the former of which have long dominated quantum chemistry.^6−8^ The idea in both Gaussian and Slater type orbital basis sets is to describe chemistry by analytic basis functions that “look like” atomic orbitals. The true atomic orbitals can be approached in such a basis set given sufficiently many basis functions,^9^ as the corresponding expansion coefficients are optimized to minimize the total energy.^10^ The benefit of this type of approach is that a relatively compact atomic-orbital basis set usually affords at least a qualitative level of accuracy for applications, while larger basis sets can enable calculations that approach quantitative accuracy with respect to experiments.^9^

However, the accuracy of analytic basis sets is limited, and in the case of Gaussian basis sets, basis set truncation errors in the order of 1 m*E*_*h*_ are typically observed in total energies of heavy atoms.^11−13^ An alternative to employing analytical basis sets that approximate the true form of atomic radial functions is to switch to methods that use exact radial functions. The exact radial functions can be solved with fully numerical methods; such calculations have been recently reviewed in ref (9).

The general idea in fully numerical methods is to forego the chemical intuition inherent in the LCAO approach, and instead directly solve the differential equations arising from the Schrödinger equation for the unknown orbitals. In the case of single atoms, the problem reduces to the determination of the atomic radial functions and yields numerical atomic orbitals (NAOs). All fully numerical methods can be systematically made more accurate, which allows the determination of SCF total energies directly at the complete basis set (CBS) limit.

The finite difference method (FDM) is the traditional method of choice in electronic structure, and it has been employed in a number of density functional implementations for atoms.^9^ However, the method of choice for solving differential equations of various forms across various disciplines is not the FDM but the finite element method (FEM). The great benefit of FEM is that it is a variational method unlike FDM and that it is straightforward to tailor the numerical basis functions used in FEM to optimize the cost and accuracy of the solution. FEM has been applied to many kinds of problems in the quantum chemistry literature.^9^

We have recently published a free and open source^14^ program for finite element calculations on atoms^15,16^ and diatomic molecules^17,18^ called HelFEM.^19^HelFEM affords an easy way to approach CBS limit total energies for density functionals, for example, as the total energies are computed variationally within each numerical basis set, and the numerical basis sets can be systematically extended toward the CBS limit. In contrast, the traditionally used FDM does not satisfy the variational theorem and can give estimated total energies that are above or below the CBS value.

We have shown that the FEM approach used in HelFEM routinely affords sub-*μE*_*h*_ total energies for Hartree–Fock (HF) and density functional calculations with local density approximations (LDAs), generalized gradient approximations (GGAs) as well as meta-GGA functionals.^13,15−18,20−22^ We have also shown that range-separated functionals can be implemented within the same scheme.^16^

Although some atomic FDM solvers that can also handle meta-GGA functionals, such as the Atomic Pseudopotentials Engine (APE),^23^ have been reported in the literature, HelFEM was to the best of our knowledge the first FEM program to be able to perform such calculations.

So far, all of our work has employed Lagrange interpolating polynomial (LIP) shape functions for FEM, yielding a  continuous numerical basis set. In this work, we will reinvestigate the choice of the shape functions. In addition to LIPs, we will consider an analytical implementation of first-order Hermite interpolating polynomials (HIPs), and show that it affords high accuracy with compact expansions through the use of high-order polynomial basis functions and a nonuniform element grid. We find that the HIP basis affords lower energies than a LIP basis with the same total number of basis functions, and thereby can recommend the use of first-order HIPs instead of LIPs.

Moreover, we will also consider a numerical implementation of higher-order HIP basis functions solved in terms of a LIP basis with a large number of nodes. Furnished with this implementation, we demonstrate that discontinuities in the two meta-GGA ingredients—local kinetic energy density τ and density Laplacian ∇^2^*n*—can be observed in HF calculations employing small numerical basis sets not yet converged to the CBS limit. In specific, the  LIP basis yields discontinuous τ and ∇^2^*n*, the *C*^1^ HIP basis yields a cuspy τ and discontinuous ∇^2^*n*, while the  second-order HIP basis yields a smooth τ and cuspy ∇^2^*n*.

However, we also demonstrate that when a large enough numerical basis set that reproduces the CBS limit is used, both τ and ∇^2^*n* come out smooth from both HF calculations as well as calculations with τ-dependent meta-GGA functionals.

The layout of this work is as follows. Next, in section 2, we will discuss the theory of FEM (section 2.1) and the choice of the basis polynomials used as the shape functions (section 2.2). The results are discussed in section 3. We discuss how numerical instabilities in the evaluation of the numerical basis functions near the nucleus can be avoided through the use of high-order Taylor expansions (section 3.1), show that HIPs afford excellent results with nonuniform grids and large numbers of nodes and overperform LIPs (section 3.2), analyze the effect of HIPs in calculations with a selection of meta-GGA density functional approximations (section 3.3), and examine their potential benefits for building NAO basis sets (section 3.4). The article concludes in a summary and discussion in section 4. Atomic units are used throughout, unless specified otherwise.

## 2 Theory

### 2.1 Finite Element Method

The employed finite element formalism has been extensively discussed in refs (15 and 24), but will be briefly summarized for completeness as we revisit the choice of the shape functions in this work. The radial functions for spin σ are expanded in the numerical basis set as
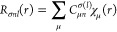
1where χ_μ_(*r*) are the numerical basis functions, which in turn are given by
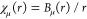
2The shape functions *B*_μ_(*r*) in eq 2 are piecewise polynomials defined in terms of *N*_elem_*elements*, each element *i* ranging from *r* = *r*_*i*_ to *r* = *r*_*i*+1_; the shape functions therefore have finite support. Note that in order to avoid eq 2 diverging at the nucleus, all shape functions are required to vanish at the origin, *B*_μ_(*r*) → 0 for *r* → 0, and this is accomplished by removing the shape function describing the function value at the nucleus.^15^ Because of this removal, all the remaining numerical basis functions become well-defined with χ_μ_(*r*) → *B*_μ_^′^(0) when *r* → 0.

The finite element grid defines the values {*r*_*i*_}; the “exponential grid” of ref (15)
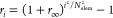
3is used in the present work. The parameter *r*_∞_ in eq 3 is the *practical infinity* employed in the calculation, beyond which all orbitals vanish; the default value^15^*r*_∞_ = 40*a*_0_ is used for all calculations in this work. The parameter *z* in eq 3 controls the structure of the element grid: the larger *z* is, the more points are placed close to the nucleus. The value *z* = 2 has been found to afford results with excellent accuracy in HF calculations,^15^ and the value *z* = 2 is also used in the present work unless specified otherwise.

### 2.2 Basis Polynomials

Three types of shape functions will be investigated: Lagrange interpolating polynomials (LIPs), as well as Hermite interpolating polynomials (HIPs) of the first and second orders. The shape functions are expressed within each element *r* ∈ [*r*_*i*_, *r*_*i*+1_] in terms of a primitive coordinate *x* ∈ [−1, 1] obtained with the transformation
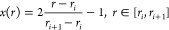
4Integrals are computed over the elements by quadrature with *N*_quad_ points, and all calculations are converged to the quadrature limit. A Chebyshev quadrature rule transformed to unit weight factor is employed for this purpose, as it provides nodes and weights in easily computable analytical form;^25^ note that the rule does not place any points at the element boundaries.

#### 2.2.1 Lagrange Interpolating Polynomials

The LIP basis was chosen as the default in HelFEM in previous works.^15,17^ The LIP basis is defined by a set of nodes {*x*_*i*_}_*i* = 1_^*N*_nodes_^ satisfying *x*_1_ = −1 < *x*_2_ < ··· < *x*_*N*_nodes__ = 1 as
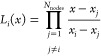
5LIPs satisfy the important property
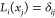
6Because of eq 6, the coefficient of the *i*th LIP *L*_*i*_(*x*) in the expansion of any given function *f*(*x*)
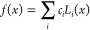
7is simply given by the value of the function at the corresponding node *f*(*x*_*i*_)
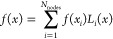
8as is easily seen by evaluating eq 7 at *x* = *x*_*j*_.

Let us now consider the continuity of the representation across element boundaries. The continuity is guaranteed, since the rightmost node *x*_*N*_nodes__ = 1 of the left-hand element coincides with the leftmost node *x*_1_ = −1 of the right-hand element; these two LIPs are thus identified as pieces of the same shape function.

Boundary conditions at the nucleus and at infinity are handled by removing the first and last numerical basis function, respectively; this ensures that the wave function vanishes at *r*_∞_ and that *B*_μ_(*r*)/*r* does not diverge at the nucleus.^15^

The Runge instability that arises for large numbers of uniformly placed nodes is avoided by choosing the nodes with the Gauss–Lobatto quadrature formula. This enables the use of extremely high-order LIPs with favorable accuracy properties,^15^ as will also be demonstrated later in this work; 15-node LIPs were chosen as the default in ref (15).

Although LIPs are only  functions and thereby do not guarantee derivatives to be continuous across element boundaries, we have demonstrated in a variety of studies performed at the density functional and HF levels of theory^13,15−18,20−22^ that the total energy converges smoothly to the complete basis set limit when more elements are added in the calculation. The rationale for this behavior is that the kinetic energy term in the Hamiltonian imposes penalties on discontinuous derivatives across boundaries.^15^ We will investigate discontinuities across element boundaries in detail in section 3.3.

#### 2.2.2 Analytic First-Order Hermite Interpolating Polynomials

First-order HIPs, which are  continuous and explicitly guarantee the continuity of the first derivative across element boundaries, can be expressed in terms of LIPs as
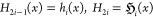
9
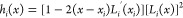
10
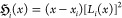
11

It is easy to see that these functions satisfy the properties
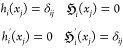
12As can be seen from eq 12 in analogy to the discussion on LIPs and eq 6 in section 2.2.1, odd-numbered functions (*h*_*i*_) carry information on the function value at the corresponding node, only, while the even-numbered functions () carry information only on the derivative at the corresponding node
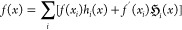
13

Nodes at the element boundaries are again used to glue the shape functions representing function values or derivatives together at each side of the boundary. The first *h*_*i*_ is removed to satisfy the boundary condition at the nucleus, while the first  describes the electron density at the nucleus.^15^ To satisfy the boundary condition at the practical infinity *r*_∞_, the last *h*_*i*_ is removed such that the radial function vanishes at *r* = *r*_∞_. If the last  is removed, as well, then the radial function’s derivative will also vanish at *r* = *r*_∞_; see section 3.4 for a case study.

Although HIPs were already examined in ref (15), the work had two deficiencies. First, in contrast to this work (eq 9), the implementation of ref (15) employed a primitive polynomial representation (*x*^*i*^ instead of *L*_*i*_(*x*)) and thereby is numerically unstable for large numbers of nodes. Second, and more importantly, the matching across element boundaries did not correctly take into account the ramifications of nonuniform elements: the coordinate scaling r → *Lr* in eq 4 that results in derivatives scaling as d^*n*^/dr^*n*^ → *L*^–*n*^d^*n*^/dr^*n*^ was not taken into account in the implementation, causing the poor results observed for nonuniform grids. Note that in order to make the derivatives match on the boundaries, one must scale the basis functions describing *n*:th derivatives at the nodes *f*_*i*_^(*n*)^(*x*_*j*_) = δ_*ij*_ by *L*^*n*^ to obtain *f*^(*n*)^(*r*) = 1 at the element boundaries. As will be shown in section 3.2, HIPs in fact afford better accuracy than LIPs with the same total number of basis functions also when nonuniform grids are employed, although either basis can be used to reach the CBS limit (sections 3.3 and 3.4).

#### 2.2.3 Numerical Hermite Interpolating Polynomials

For comparison and simplicity, we have also implemented higher-order HIPs numerically. The HIPs can be solved numerically in terms of LIPs from the linear system of equations corresponding to generalizations of eq 13 to higher orders. For instance, the expansion for the second order reads
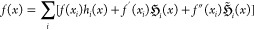
14and the functions *h*_*i*_(*x*),  and  satisfy the equations
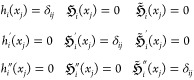
15where *i* and *j* are node indices, *i*, *j* ∈ [1, *N*_nodes_^HIP^]. In our implementation, the general *n*:th order HIP basis functions (for example, eq 14 for *n* = 2), which guarantees  continuity, is re-expressed in terms of LIPs with *N*_nodes_^LIP^ = (*n* + 1) *N*_nodes_^HIP^ as ***H***(*x*) = ***L***(*x*)***T***, where ***H*** are the HIP basis functions, ***L*** are the underlying LIP functions, and ***T*** is the transformation matrix. Gauss–Lobatto nodes are used for both the *N*_nodes_^HIP^ HIP nodes as well as the *N*_nodes_^LIP^ LIP nodes, as discussed in section 2.2.1. The transformation matrix ***T*** is solved numerically by inverting eq 15.

## 3 Results

### 3.1 Numerically Stable Evaluation of Basis Functions

Before proceeding with electronic structure calculations, it is worthwhile to discuss the stable evaluation of the numerical basis functions in regions close to the nucleus. Exploratory calculations performed as part of this work showed that in some cases, calculations with otherwise well-behaved meta-GGA functionals failed to reach SCF convergence and showed extremely large orbital gradients. We found the difference between well-behaved and ill-behaved calculations to center around the few closest quadrature points to the nucleus that had extremely large values for τ, and we were able to remove this issue by reformulating the numerical basis functions in a more stable manner.

Taylor expanding the basis functions of eq 2 around *r* = 0 shows that they are in principle well-behaved everywhere as
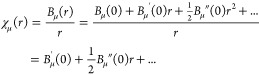
16where *B*_μ_(0) = 0,^15^ even though the evaluation of *B*_μ_(*r*)/*r* and its derivatives is unstable for small *r*. For this reason, we employ Taylor expansions to evaluate χ_μ_(*r*) and its derivatives close to the nucleus, that is, for *r* < *R*.

Since all matrix elements are evaluated by quadrature, the use of eq 16*is not an approximation*. Instead, eq 16 amounts to a redefinition of the numerical basis functions near the nucleus
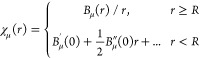
17since all matrix elements are evaluated with respect to the numerical basis defined by eq 17. The Taylor expansion technique is therefore fully compatible with the variational approach pioneered in ref (15).

Even though eq 17 constitutes a valid definition for the numerical basis regardless of the order of the used Taylor expansion or the employed switch-off value *R*, it is best for accuracy and numerical stability if the switchoff between the analytic expression and the Taylor expansion is as smooth as possible.

We determine the optimal switching point *R* by maximizing the mutual compatibility of the two definitions for the basis functions and their derivatives at *R*. We measure the agreement with the metric
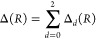
18
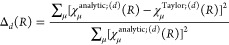
19which simultaneously considers the compatibility of the numerical basis functions themselves as well as that of their first two derivatives.

Since the evaluation of primitive polynomials *f*(*r*) = ∑_*n*_*c*_*n*_*r*^*n*^ is ill-behaved in general, and the analytical expression χ_μ_(*r*) = *B*_μ_(*r*)/*r* is only well-behaved at non-negligible *r*, we will only consider values of *R* in the region 0 ≤ *R* ≤ *r*_2_, where *r*_2_ is the position of the second node defining the shape functions *B*_μ_(*r*), the first node always being located at *r*_1_ = 0 and the corresponding basis function removed to satisfy the boundary condition *B*_μ_(0) = 0.

**Figure 1:**
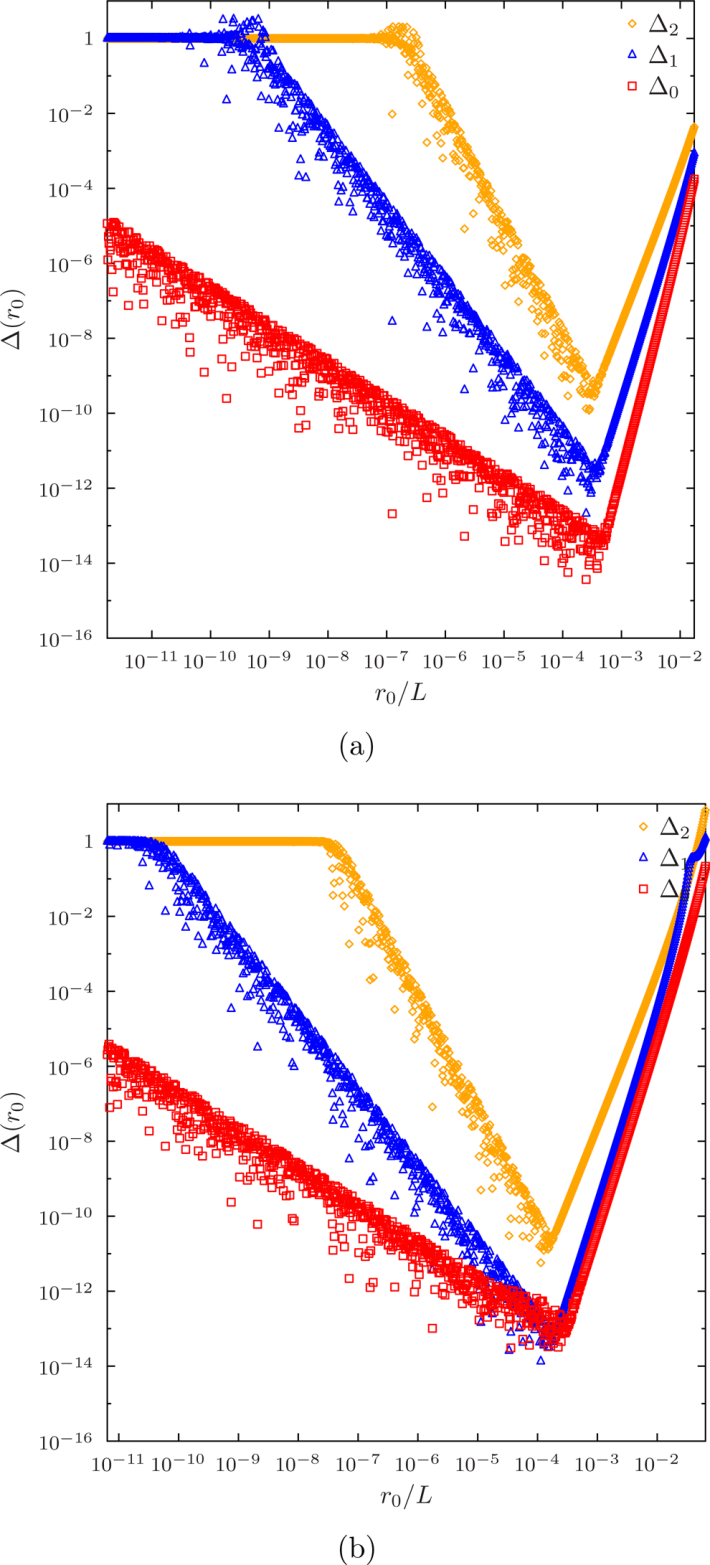
Error (eq 18) in approximate Taylor expansions of basis functions and their derivatives (eq 16) as a function of the switchoff radius *r*_0_ in terms of the length *L* of the first radial element. (a) Data for a sixth order Taylor expansion in 15-node LIP basis with 5 radial elements. The first non-nuclear node is at *r*/*L* ≈ 0.017377. (b) Data for a sixth order Taylor expansion in 8-node HIP basis with 5 radial elements. The first non-nuclear node is at *r*/*L* ≈ 0.064130.

With the restriction to 0 ≤ *R* ≤ *r*_2_, the fitting problem is well-behaved. The result of a numerical experiment for a low-order Taylor expansion is shown in Figure 1. As the analytic functions are numerically unstable for small *R*, while the Taylor expansion becomes inaccurate for large *R*, Δ(*R*) has a well-defined minimum, but due to finite numerical precision, there is bound to be some noise. As expected, the optimal switching value *R* is found to the left of the first non-nuclear node, *R* < *r*_2_, and the error Δ(*R*) decreases monotonically when approaching the optimal value from the right, until it starts going back up again for *r* < *R* where the analytical expression χ_μ_(*r*) = *B*_μ_(*r*)/*r* is unstable. As the noise makes it potentially risky to pick *R* from the global minimum of Δ(*R*), we choose *R* by proceeding downhill to the left from *R* = *r*_2_.

Figure 1 also shows that the errors increase for every derivative when a low-order Taylor expansion is used, as the Taylor expansion (eq 16) for the derivatives χ_μ_^(*d*)^(*r*) with *d* ≥ 1 have fewer and lower-order terms than the expansion for the basis function χ_μ_(*r*). The total error in eq 18 is thereby dominated by errors in the second derivative χ_μ_^″^(*r*_0_) when a low-order Taylor expansion is used.

**Figure 2:**
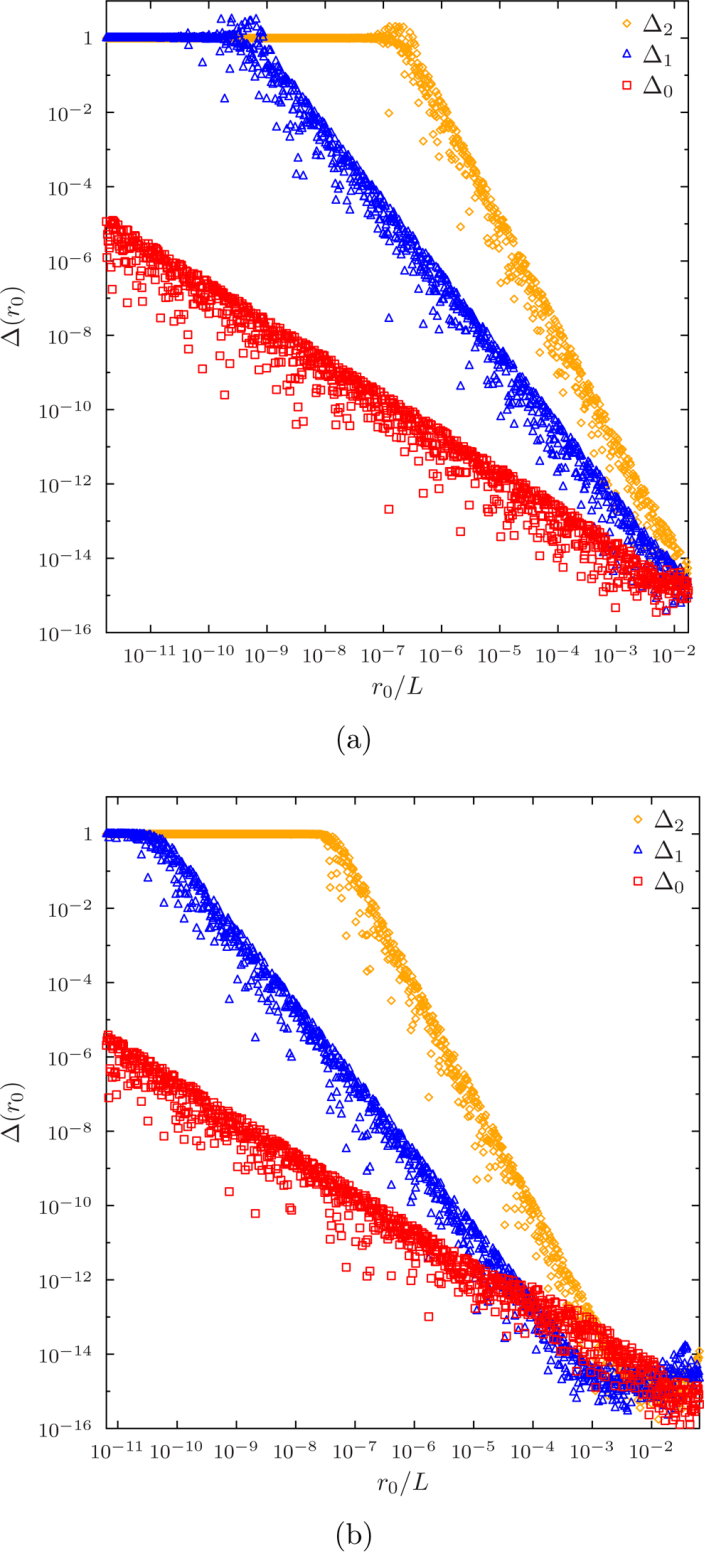
Error (eq 18) in exact Taylor expansions of basis functions and their derivatives (eq 16) as a function of the switchoff radius *r*_0_ in terms of the length *L* of the first radial element. (a) Data for a 14th order Taylor expansion in 15-node LIP basis with 5 radial elements. The first non-nuclear node is at *r*/*L* ≈ 0.017377. (b) Data for a 15th order Taylor expansion in 8-node HIP basis with 5 radial elements. The first non-nuclear node is at *r*/*L* ≈ 0.064130.

However, when the order of the Taylor series is increased, we observe that the optimal value of *R* moves to the right, and the corresponding minimal value Δ(*R*) goes down. When a Taylor expansion of an order matching that of the shape function basis *B*_μ_(*r*) is used, the optimal Δ(*R*) is practically zero, as shown in Figure 2, and the switchoff value moves all the way to the right, becoming *R* = *r*_2_.

Since the analytic derivatives of the shape functions described in section 2.2 are easy to generate to arbitrarily high orders (at present the code supports up to 20th order Taylor expansions), in the following, we employ such full-length Taylor expansions in all calculations, which is also the new default in HelFEM.

### 3.2 Supremacy of HIPs over LIPs

So far the study has used the pre-established finite element grid from ref (15). However, since the grid was optimized in ref (15) for LIPs and noble gas atoms at the HF level of theory, it is worthwhile to check whether the same grid also works well with HIPs and density functionals of various rungs. It is interesting to note that an *n*^th^ order HIP calculation (LIPs correspond to *n* = 0, first-order HIPs to *n* = 1) has
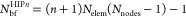
20basis functions, if the derivatives are set to zero at the practical infinity.^26^ This means that apples-to-apples comparisons of numerical basis sets for various orders is possible by choosing values for the order *n* and number of nodes *N*_nodes_ whose product yields a constant (*n* + 1) (*N*_nodes_ – 1), such as *N*_nodes_^LIP^ = 2*N*_nodes_^HIP^ – 1 for comparisons of LIPs with first order HIPs.

**Figure 3:**
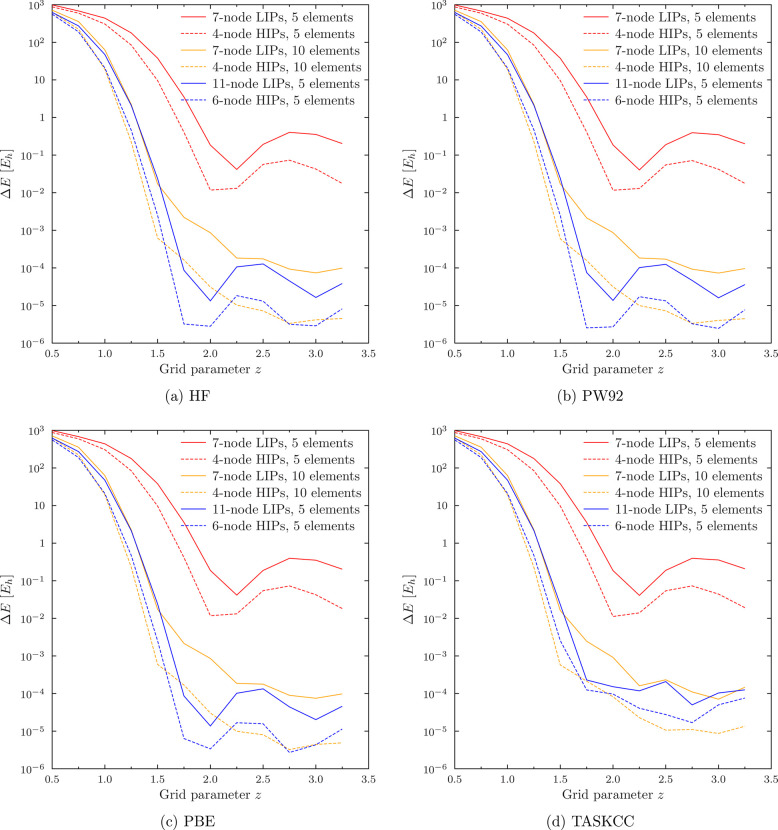
Truncation error *ΔE*(*z*) = *E*(*z*) – *E*^CBS^ in total energy of Zn as a function of the finite element grid parameter *z* (eq 3) for (a) HF, (b) PW92, (c) PBE, and (d) TASKCC. Note logarithmic *y* axis.

An illustration of the superior accuracy of the HIP basis over the LIP basis is shown in Figure 3, which studies optimal choices for the radial finite element grid to compute the total energy of Zn for such compatible choices for LIPs (*n* = 0) and the analytic first-order HIPs (*n* = 1). In addition to HF, which was used in ref (15) to determine the recommended value *z* = 2, Figure 3 also considers the 1992 LDA correlation functional by Perdew and Wang^27^ used in combination with LDA exchange^28,29^ (PW92), the 1992 GGA exchange-correlation functional by Perdew et al.^30^ (PBE), and the 2019 meta-GGA exchange functional of Aschebrock and Kümmel^31^ in combination with the meta-GGA correlation functional of Schmidt et al.^32^ (TASKCC) as suggested by Lebeda et al.;^33^ all density functionals are evaluated in HelFEM with Libxc.^34^

Figure 3 again demonstrates that the choice for the grid is extremely important, as the quality of the resulting wave function is entirely dependent on a suitable distribution of the degrees of freedom. Nonuniform grids afford similarly good results with LIPs and HIPs. Figure 3 also supports the general agreement based on Gaussian-basis calculations^35^ that the basis set requirements of HF and DFT calculations are similar: the truncation errors for HF, PW92, PBE, and TASKCC are similar in behavior and magnitude.

As Figure 3 shows, improvements of roughly an order of magnitude are possible when switching over from a LIP basis to a HIP basis with the same total number of basis functions. However, this only applies when the basis sets are limited to a low numerical order: when a large enough numerical basis set is used, either basis converges to the same total energy.

As was discussed in ref (15), the convergence of the SCF energy to the CBS limit is extremely rapid (superexponential) with the number of basis functions, when the number of nodes is also increased; note that this roughly corresponds to *hp*-adaptive FEM where both the discretization (*h*) and the order of the polynomial basis (*p*) is changed, even though our grids are merely empirically optimal. By default, HelFEM employs numerical basis functions of a high order, and the CBS limit can be routinely reached by simply adding more radial elements until the total energy does not change any more.

**Figure 4:**
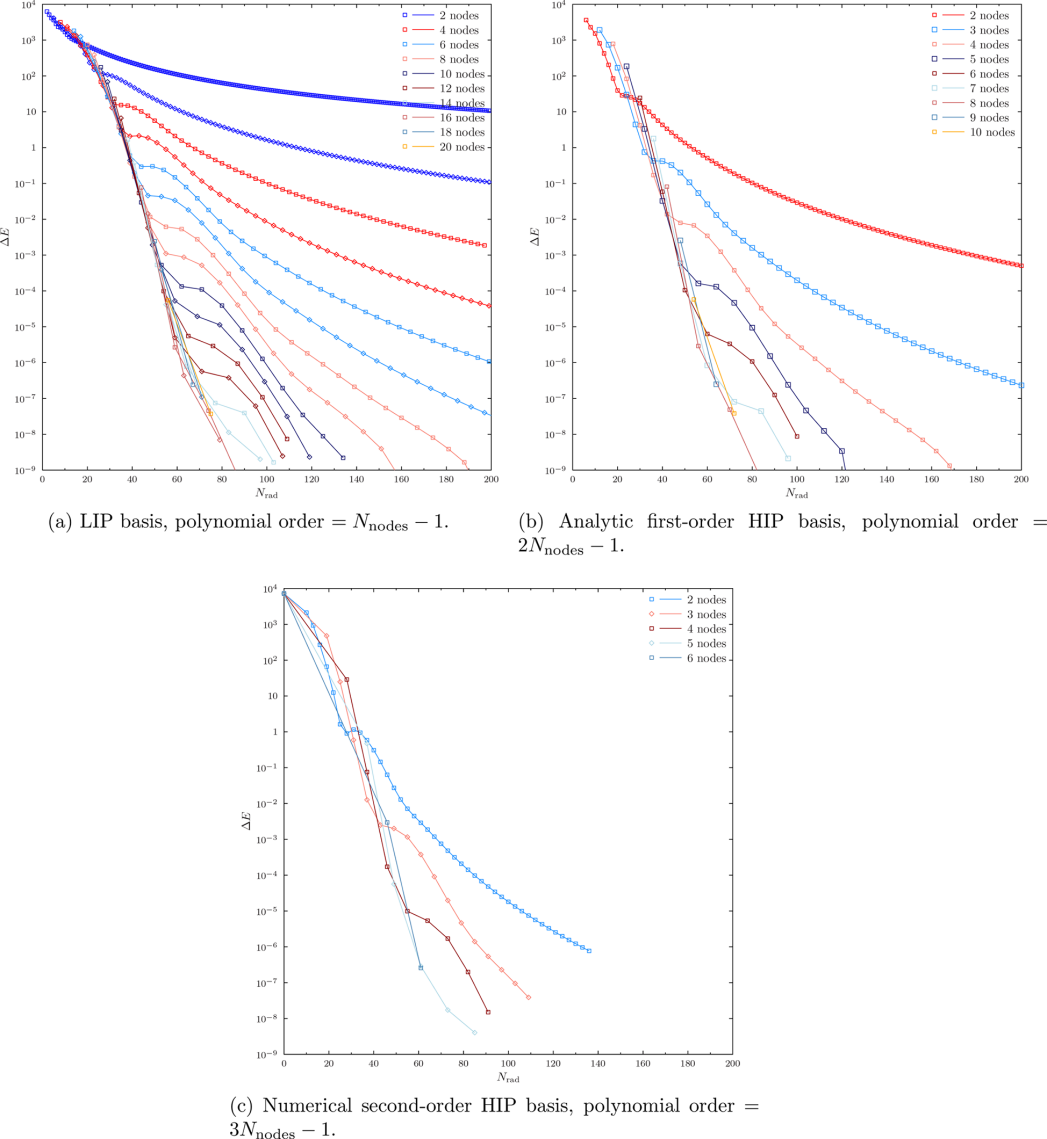
Truncation error *ΔE* = *E*–E^CBS^ in total Hartree–Fock energy of the Xe atom for (a) LIP, (b) analytical first-order HIP, and (c) numerical second-order HIP basis sets. Note logarithmic *y* axis. The CBS limit energy is *E*^CBS^ = −7232.13836387 as determined by Saito.^36^ Plots for even numbers of nodes are shown with solid lines marked with squares, as shown in the legend, while plots for odd numbers of nodes are shown with solid lines marked with diamonds, the basis having one more node than the even-numbered calculation of the same color. The colors and markers in parts b and c are chosen such that the order of the polynomial basis matches in parts a–c.

In practice, either basis (LIP or HIP) can be used to converge the total energy to the CBS limit, which is the whole point of employing fully numerical methods. At the end, it should not matter which numerical method is used to obtain results, only that the results are fully converged to the CBS limit. Following ref (15), the power of high-order numerical schemes is demonstrated by the truncation errors plotted in Figure 4 for the studied LIP and HIP methods.

We observe that the accuracy obtained with LIPs and analytical first-order HIPs, as well as numerical second-order HIPs, is highly affected by the employed polynomial order, as is demonstrated by the rapid decrease of the truncation error at fixed number of radial basis functions *N*_rad_ with increasing polynomial order of the basis. This finding was one of the main results of ref (15), but that study was limited to LIPs.

We also see great similarities between Figure 4a and 4b as well as Figure 4a and 4c: the plots for the same polynomial order with LIPs and HIPs have almost the same shape, the HIP calculation just having fewer basis functions in total, since more functions are overlaid as discussed above.

### 3.3 HIPs and Meta-GGA Functionals

Next, we will look deeper into how the numerical basis set affects the calculations with meta-GGA functionals for the exchange-correlation (xc) energy, which depend on the spin-σ local kinetic energy density
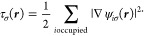
21and/or the Laplacian of the spin-σ electron density ∇^2^*n*_σ_ as
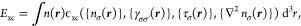
22where γ_σσ′_(***r***) = ∇*n*_σ_(***r***)·∇*n*_σ′_(***r***) is the reduced gradient and ϵ_xc_ is the used density functional approximation (DFA) that models the energy density per particle.

#### 3.3.1 Exact Results for the Hydrogen Atom

A simple test is offered by the hydrogen atom, whose exact nonrelativistic Born–Oppenheimer ground state is ψ(***r***) = π^–1/2^e^–*r*^. As the wave function is spherically symmetric, we will examine τ(*r*) and [∇^2^*n*](*r*) which represent τ(***r***) and ∇^2^*n*(***r***) integrated over all angles:
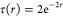
23and
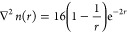
24Note that eq 24 is negative for *r* < 1, diverging to – ∞ for *r* → 0, and is positive for *r* > 1.

Before proceeding to the CBS limit, it is useful to study results that are not fully converged with respect to the basis set. We use HF for this demonstration, as it is exact for the H atom. We employ 5 radial elements and study a 7-node LIP basis (29 radial basis functions, *E* = −0.4999993*E*_*h*_), a 4-node analytic first-order HIP basis set (29 radial basis functions, *E* = −0.4999999*E*_*h*_), and a 3-node numerical second-order HIP basis set (29 radial basis functions, *E* = −0.5000000*E*_*h*_) that is expressed in terms of an underlying 9-node LIP basis.

**Figure 5:**
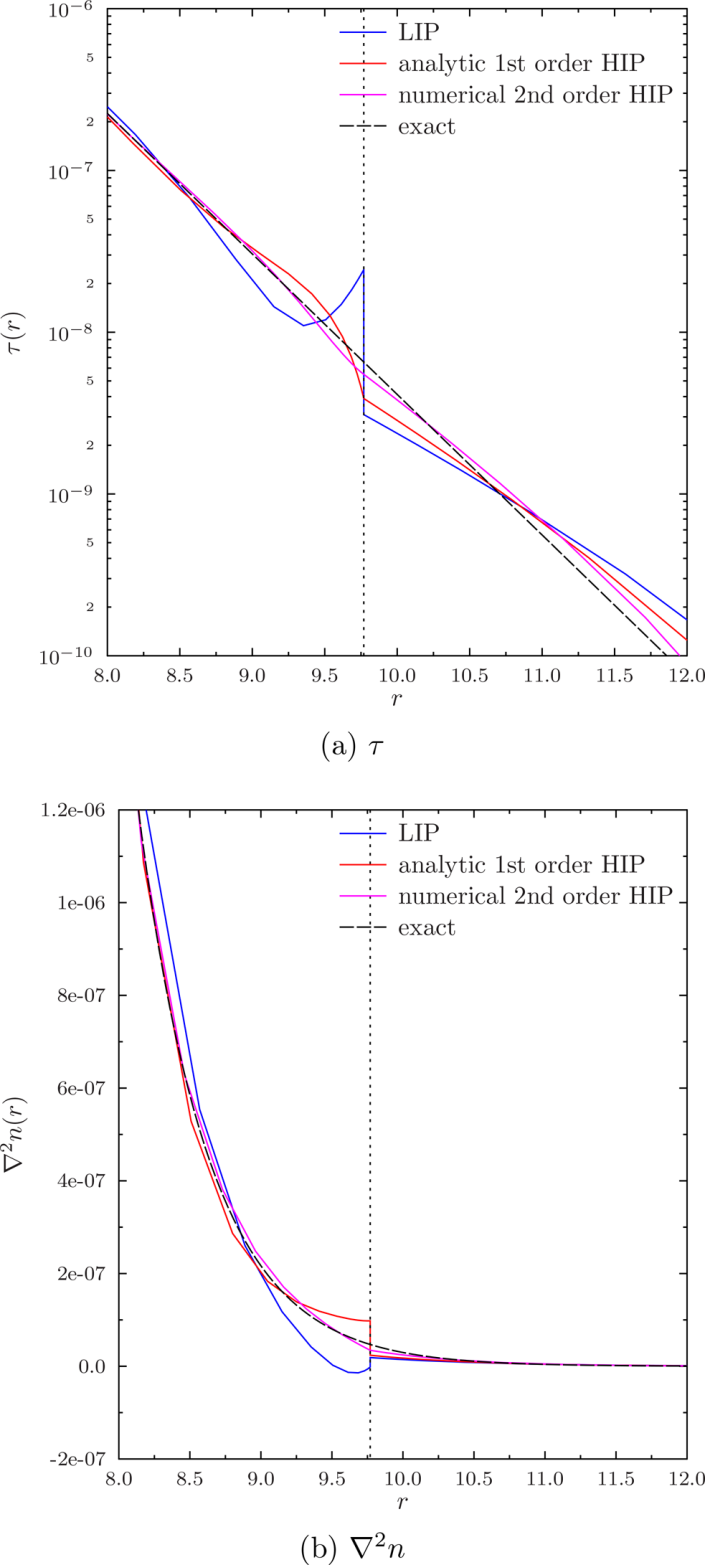
Closeup of discontinuities in τ and ∇^2^*n* of the ground state of the hydrogen atom in calculations not converged to the CBS limit. The exact values are computed with eq 23, respectively, and the boundary between the fourth and fifth radial elements is shown with the vertical dotted line.

We observe that there are discontinuities in τ and ∇^2^*n* between the fourth and fifth radial elements, shown in Figure 5, even though all the numerical energies are very close to the exact value *E* = −0.5*E*_*h*_. As can be seen from Figure 5a, the LIP basis shows a step discontinuity already in τ, which is also accompanied by a step discontinuity in ∇^2^*n* as seen from Figure 5b. The first-order analytic HIP curve shows a cusp in τ, that is, a discontinuity of the first derivative of τ, while ∇^2^*n* is still stepwise discontinuous. The numerical second-order HIP basis, on the other hand, produces a smooth τ but still has a noticeable cusp in ∇^2^*n* at the element boundary.

However, these issues go away when the size of the numerical basis set is increased. In the rest of this section, we will consider 15-node LIPs, 8-node analytic first-order HIPs, and 5-node numerical second-order HIPs that are expressed in terms of a 15-node LIP basis, all with five radial elements. Each of these basis sets reproduces a HF total energy of −0.5000000*E*_*h*_.

**Figure 6:**
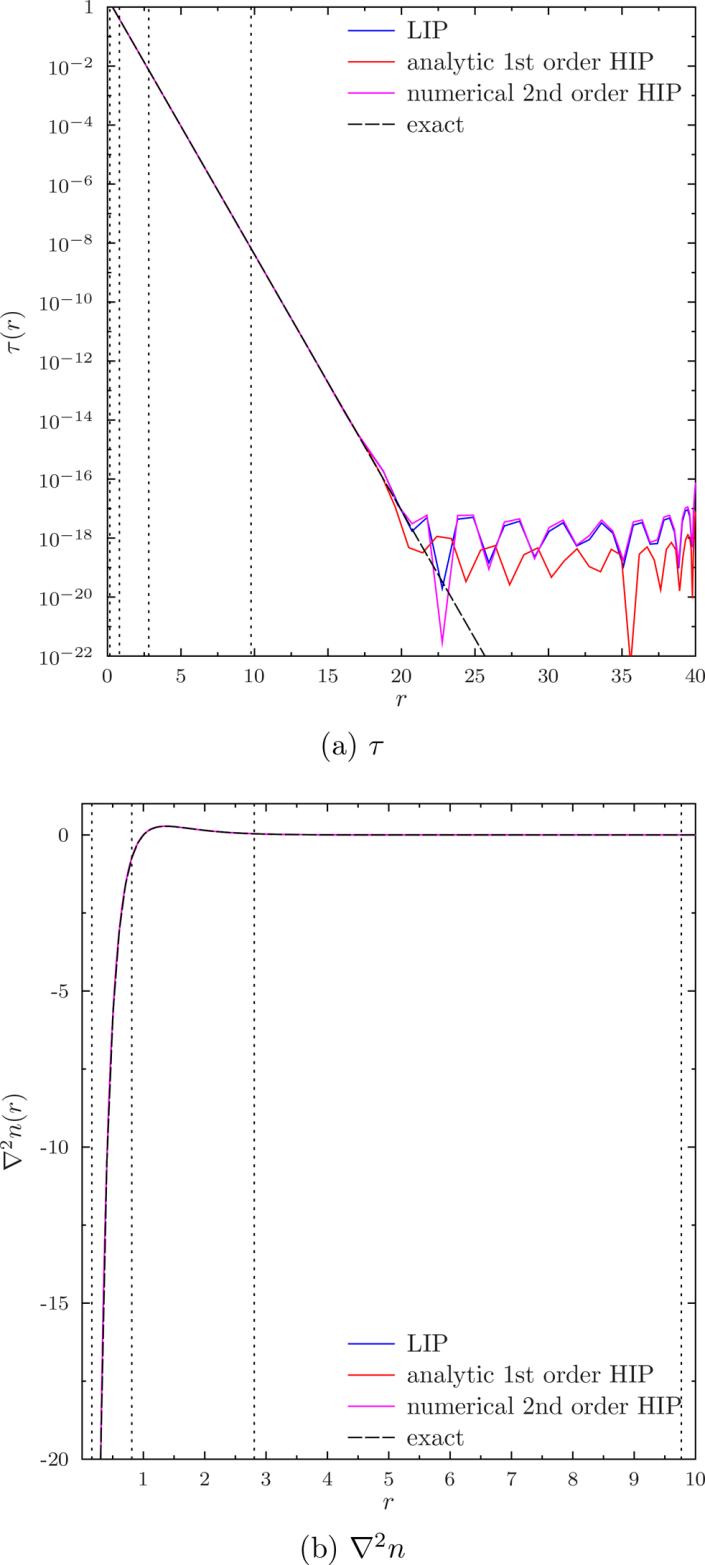
τ and ∇^2^*n* of the ground state of the hydrogen atom, computed with HF with either a 15-node LIP basis, an 8-node analytic first-order HIP basis set, or a 5-node numerical second-order HIP basis set. Five radial elements were used in all calculations. The exact values are computed with eqs 23 and 24, respectively, and the boundaries between the radial elements are shown with the vertical dotted lines.

We have previously argued that the energy minimization involved in the variational theorem of self-consistent field theory should ensure a smooth wave function, even if the underlying numerical basis set does not guarantee explicit continuity.^15^ This indeed does appear to be the case: we see that both τ and ∇^2^*n* are successfully reproduced for the hydrogen atom by the larger numerical basis sets without problems, as shown in Figure 6a for τ and Figure 6b for ∇^2^*n*.

#### 3.3.2 Self-Consistent Calculations

We have now established that all three numerical basis sets are able to reproduce τ and ∇^2^*n* in HF calculations. We note that we have recently analyzed a thorough selection of density functionals in ref (37) and found that many recent meta-GGAs are numerically ill-behaved at fixed density. We wish to continue this work here by studying how the smoothness of the numerical basis set used to represent the orbitals can affect the behavior of the density functional when the density is relaxed. The form of the numerical basis may be important in calculations with meta-GGA functionals, since the functionals depend explicitly on τ and/or ∇^2^*n* through eq 22.

We note that τ-dependent functionals form an overwhelming majority of available meta-GGAs,^34^ and that many of the few available Laplacian dependent functionals were found ill-behaved in ref (37). For this reason, we will only discuss τ-dependent functionals in this work. We have previously described successful calculations with τ-dependent meta-GGA functionals with LIP basis sets,^15,17,22^ and we will critically study such calculations here.

We begin by the general note that we have observed that calculations with meta-GGAs often fail when the numerical basis is not large enough for CBS limit quality, such as when employing only one or two radial elements instead of the five elements employed to obtain these results; similar observations were made with both LIPs and the first- and second-order HIPs. Such convergence problems can be understood through the discussion on the exact ground state in section 3.2: a basis set that is not sufficiently flexible can produce spurious cusps or oscillations in the optimal τ (example in Figure 5), whereas the optimal τ in a more complete basis is likely smoother and thereby easier to find. Meta-GGA calculations should therefore use numerical basis sets that can reproduce the CBS limit energy for HF at the very least.

Our selection of meta-GGA functionals starts out with the TPSS functional of Tao et al.^38^ and TASKCC.^31,32^ The r^2^SCAN functional^39,40^ has been found ill-behaved in a recent fully numerical study;^41^ we also found r^2^SCAN to be ill-behaved at fixed electron density in ref (37), and we choose to add it to our present study. For completeness, we also study a number of semiempirical meta-GGA functionals, which might present other types of issues. We limit ourselves to functionals that did not appear problematic at fixed electron densities.^37^

Our selection includes two major families of modern semiempirical density functionals. First, we have the Minnesota family composed of the M05,^42^ M05-2X,^43^ M06,^44^ M06-2X,^44^ M11,^45^ MN12-SX,^46^ and MN15^47^ hybrid functionals as well as their local versions M06-L,^48^ M11-L,^49^ MN12-L,^50^ and MN15-L,^51^ respectively, accompanied by the most recent additions: the 2017 revM06-L functional,^52^ the 2018 revM06 functional,^53^ 2019 revM11^54^ functional, and the 2020 M06-SX functional.^55^ Second, the Berkeley family is formed by the ωB97X-V^56^ hybrid GGA, the B97M-V^57^ meta-GGA, and ωB97M-V^58^ hybrid meta-GGA functionals by Mardirossian and Head-Gordon. As it is well-known that the Vydrov–van Voorhis nonlocal correlation^59^ (VV10) does not change the electron density significantly,^60^ we will only consider ωB97X-V, B97M-V, and ωB97M-V without the nonlocal correlation part, which we denote as ωB97X-noV, B97M-noV, and ωB97M-noV, respectively.

The comparison of the Minnesota and Berkeley meta-GGA functionals is interesting, as the functionals utilize similar ingredients: τ dependence is expressed in terms of the normalized variable −1 ≤ *w*_σ_ ≤ 1 given by
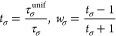
in all of the above functionals with the exceptions of M05, M06, M06-2X, M06-SO, M06-L, revM06, and revM06-L.

The present finite element study of these functionals is motivated by the finding of Mardirossian and Head-Gordon^61^ that many Minnesota functionals converge slowly to the CBS limit; yet many of the presently considered Minnesota functionals were published after ref (61). We also found surprisingly large Gaussian basis set truncation errors in exploratory FEM calculations with the M06-L^48^ functional in a recent study.^62^

All total energies are summarized in Table 1. The TPSS, TASKCC or r^2^SCAN functionals pose no issues for the H atom, and converge to the same total energies in all three numerical basis sets.

**Table 1:** Total Energies for H Atom with Various Numerical Basis Sets^a^

method	LIP	first order HIP	second order HIP
HF	–0.5000000	–0.5000000	–0.5000000
TPSS	–0.5002355	–0.5002355	–0.5002355
TASKCC	–0.5001730	–0.5001730	–0.5001730
r^2^SCAN	–0.5001732	–0.5001732	–0.5001732
M05	–0.4999407	–0.4999432	–0.4999372
M05-2X	N/C	N/C	N/C
M06	–0.5012906	–0.5012928	–0.5012897
M06-2X	N/C	N/C	N/C
M06-SX	–0.4856891	–0.4856891	–0.4856891
M06-L	–0.5049877	–0.5049876	–0.5049872
revM06	–0.4978698	–0.4978698	–0.4978698
revM06-L	–0.5000720	–0.5000720	–0.5000720
M08-SO	N/C	–0.5039712	–0.5039475
M08-HX	–0.5039980	–0.5039981	–0.5039979
M11	–0.4998235	–0.4998251	–0.4998216
revM11	–0.5023467	–0.5023467	–0.5023467
M11-L	–0.5128008	–0.5127346	–0.5126698
MN12-SX	–0.4970768	–0.4970769	–0.4970768
MN12-L	–0.4923232	–0.4923232	–0.4923232
MN15	–0.4997453	–0.4997453	–0.4997453
MN15-L	–0.4965988	–0.4965988	–0.4965988
ωB97X-noV	–0.5053272	–0.5053272	–0.5053272
B97M-noV	–0.5061077	–0.5061077	–0.5061077
ωB97M-noV	–0.4992064	–0.4992064	–0.4992064

a N/C: Calculation did not reach SCF convergence in 500 iterations.

Continuing to the semiempirical Minnesota functionals, starting out with M05, we observe that the results for M05 are not converged to the CBS limit, as is also obvious from the different total energies obtained in the various calculations in Table 1. The poor convergence is partly explained by strong oscillations observed in the τ and ∇^2^*n* of the solutions (see Supporting Information), which speak to the numerical ill-behavedness of the functional. SCF calculations for M05-2X, in turn, fail in all basis sets, suggesting numerically ill-behavedness also in this functional.

M06 is not converged to the CBS limit, and large oscillations in ∇^2^*n* are again observed in the SCF solutions; the same observations also apply to M06-L. M06-2X is too unstable to reach SCF convergence like M05-2X. In contrast, the recent revM06, revM06-L, and M06-SX functionals appear well-behaved: the calculations in all three basis sets converge to the same total energy and τ and ∇^2^*n* appear smooth.

M08-SO fails to yield a converged SCF solution in the LIP basis, and as evidenced by the difference of the first-order and second-order HIP results, is also not converged to the CBS limit; large oscillations in ∇^2^*n* are observed in the corresponding solutions. M08-HX is almost at the CBS limit, as the total energies reproduced by the three basis sets agree to within 0.2*μE*_*h*_. However, sharp nonphysical behavior is observed in ∇^2^*n* of the solution.

M11 and M11-L are also characterized by lack of CBS convergence and sharp oscillations in ∇^2^*n*. In contrast, revM11 is better behaved, but features a bump and shoulder in ∇^2^*n* that do not exist in revM06 or revM06-L that are much closer to the exact ∇^2^*n*.

MN12-SX, MN12-L, MN15, and MN15-L are well-behaved for the H atom: all three basis sets yield the same total energy and the resulting ∇^2^*n* is smooth. MN12-L and MN12-SX feature a shoulder that is similar to, but less pronounced than that for revM11.

All members of the Berkeley family are well-behaved: ωB97X-noV, B97M-noV, and ωB97M-noV all converge smoothly to the CBS limit with the studied basis sets and show no sharp features in ∇^2^*n*, although the two meta-GGAs reproduce ∇^2^*n* which is slightly different from the exact value.

### 3.4 Potential Implications for Construction of NAO Basis Sets

In this section, we investigate whether the control of derivatives afforded by HIPs could be useful for NAO basis set construction. A key difference between NAO basis sets and analytical basis sets commonly used in LCAO discussed in the Introduction is that NAO basis sets have finite support:^9^ the NAO basis functions vanish outside a given cutoff radius *R* from the nucleus—χ_α_(***r***) = 0 for *r* > *R*—which affords significant sparsity in large systems that is commonly exploited for performance benefits. The established approach to build NAO basis sets is to use FDM with various confinement potentials,^63−76^ as it is desirable that both the radial function (eq 2) and its derivative
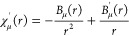
25go smoothly to zero when approaching the cutoff radius *R*, as large derivatives χ_μ_^′^(*r*) close to the cutoff *r* ≈ *R* are problematic for the evaluation of molecular integrals by quadrature.

In FEM, the LIP basis allows direct control on the boundary value *B*_μ_(*r*_∞_), which also appears in the first term of eq 25. A key feature of HIPs is that they allow explicit control also on the boundary conditions for derivative(s), such as the second term of eq 25. We will therefore investigate whether the added control on the second term provided by the HIP basis is useful for building NAO basis sets by studying the magnesium atom with LIP and HIP basis sets with various values for the practical infinity *r*_∞_. In these calculations, we employ 5 radial elements with 15-node LIPs or 8-node HIPs and the PBE functional.^30,77^

**Figure 7:**
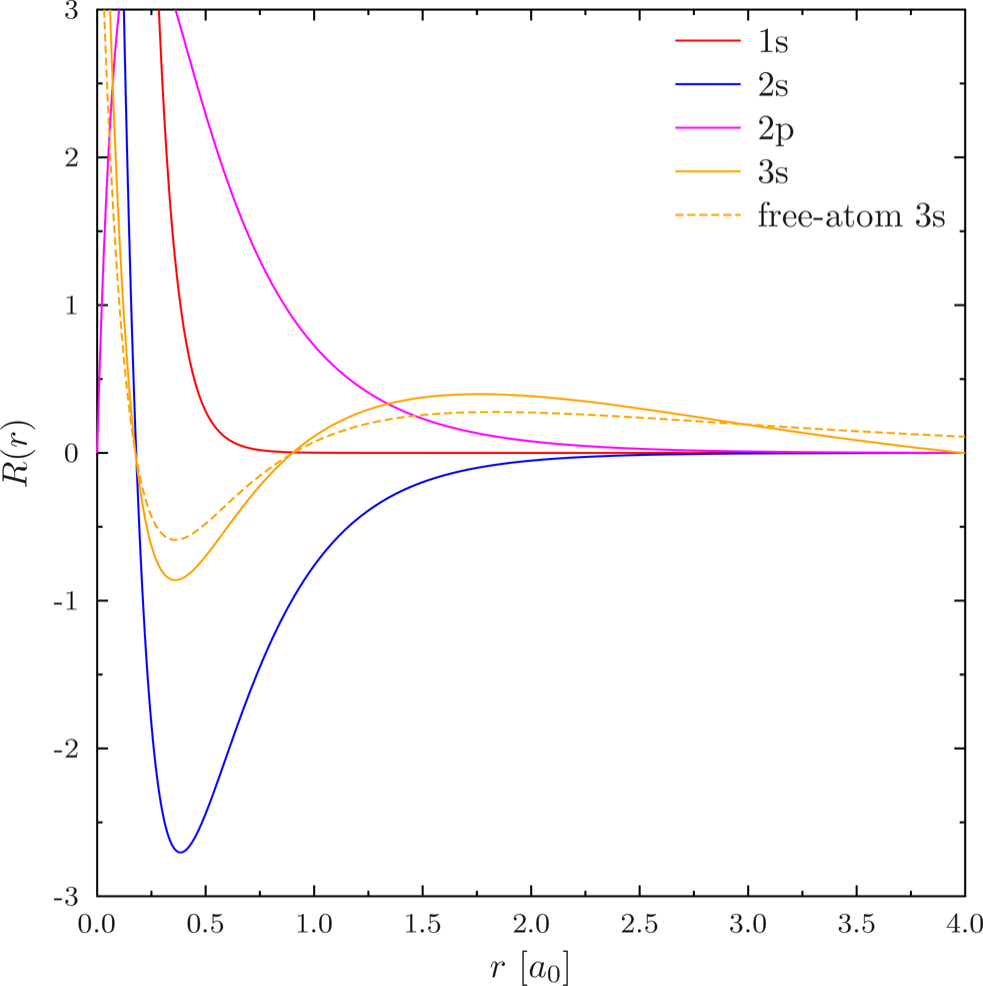
Radial orbitals of Mg with a 8-node HIP basis with 5 radial elements, the PBE functional and *r*_∞_ = 4*a*_0_. The free-atom 3s orbital (*r*_∞_ = 40*a*_0_) is also shown as reference. The difference between the solutions for *B*_μ_^′^(*r*_∞_) ≠ 0 and *B*_μ_^′^(*r*_∞_) = 0 is too small to be seen in the plot.

**Figure 8:**
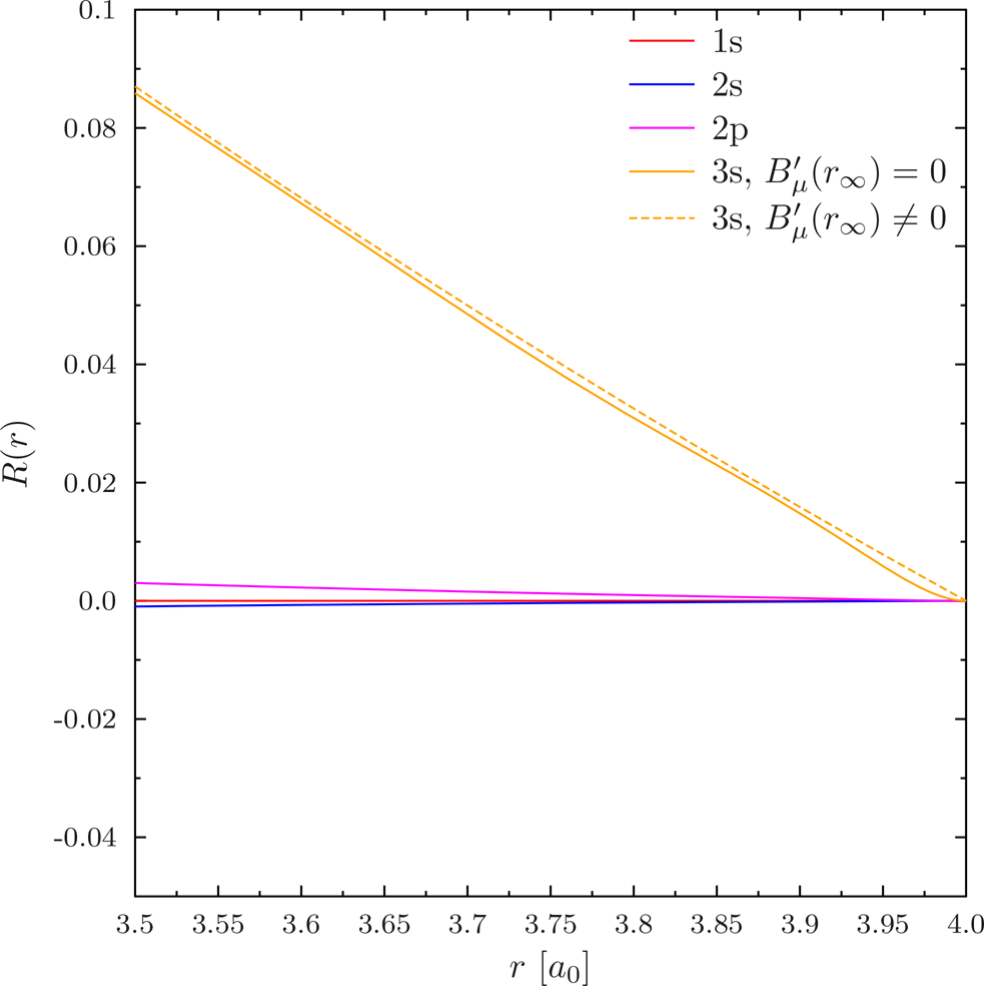
Closeup of radial orbitals of Mg shown in Figure 7, focused in the region near *r*_∞_ = 4*a*_0_. The difference between the solutions for *B*_μ_^′^(*r*_∞_) ≠ 0 and *B*_μ_^′^(*r*_∞_) = 0 is visible.

In this demonstration, we will consider LIPs and two types of calculations with HIPs: one where a finite derivative is allowed at *r*_∞_ (yielding analogous results to the LIP basis), and another one where the derivative is forced to vanish at *r*_∞_. The value *r*_∞_ = 4*a*_0_ suffices for this demonstration. The resulting radial 1s, 2s, 2p, and 3s orbitals are shown in Figure 7; only the 3s orbital turns out to differ significantly from the free atom. The issue for NAO basis set construction is that the 3s orbital develops a near-constant slope for large *r* in the constrained atom, all the way up to *r*_∞_. The derivative can, however, be made to vanish at *r*_∞_ as demonstrated by Figure 8.

But, as is obvious by the oscillations of the *B*_μ_^′^(*r*_∞_) = 0 curve in Figure 8, this calculation is not converged to the CBS limit even though the *B*_μ_^′^(*r*_∞_) ≠ 0 calculation is, as is demonstrated in Table 2. As these data show, imposing the zero-derivative condition appears to make the calculation sensitive to the finite element representation.

**Table 2:** Convergence of the PBE Total Energy of Mg with *r*_∞_ = 4*a*_0_, either Allowing a Finite Derivative of the Radial Function at the Practical Infinity or Disallowing It^a^

*N*_elem_	*N*_bf_	*E*[*B*_μ_^′^(*r*_∞_) ≠ 0]	*E*[*B*_μ_^′^(*r*_∞_) = 0]
1	13	–198.854894880	–198.818748501
2	27	–199.616629182	–199.610609917
3	41	–199.616629885	–199.611532191
4	55	–199.616629940	–199.612275491
5	69	–199.616629942	–199.612844761
10	139	–199.616629942	–199.614364754
20	279	–199.616629942	–199.615382891
40	559	–199.616629942	–199.615974661
80	1119	–199.616629942	–199.616293874

a The default finite element grid with *z* = 2 is employed.

**Figure 9:**
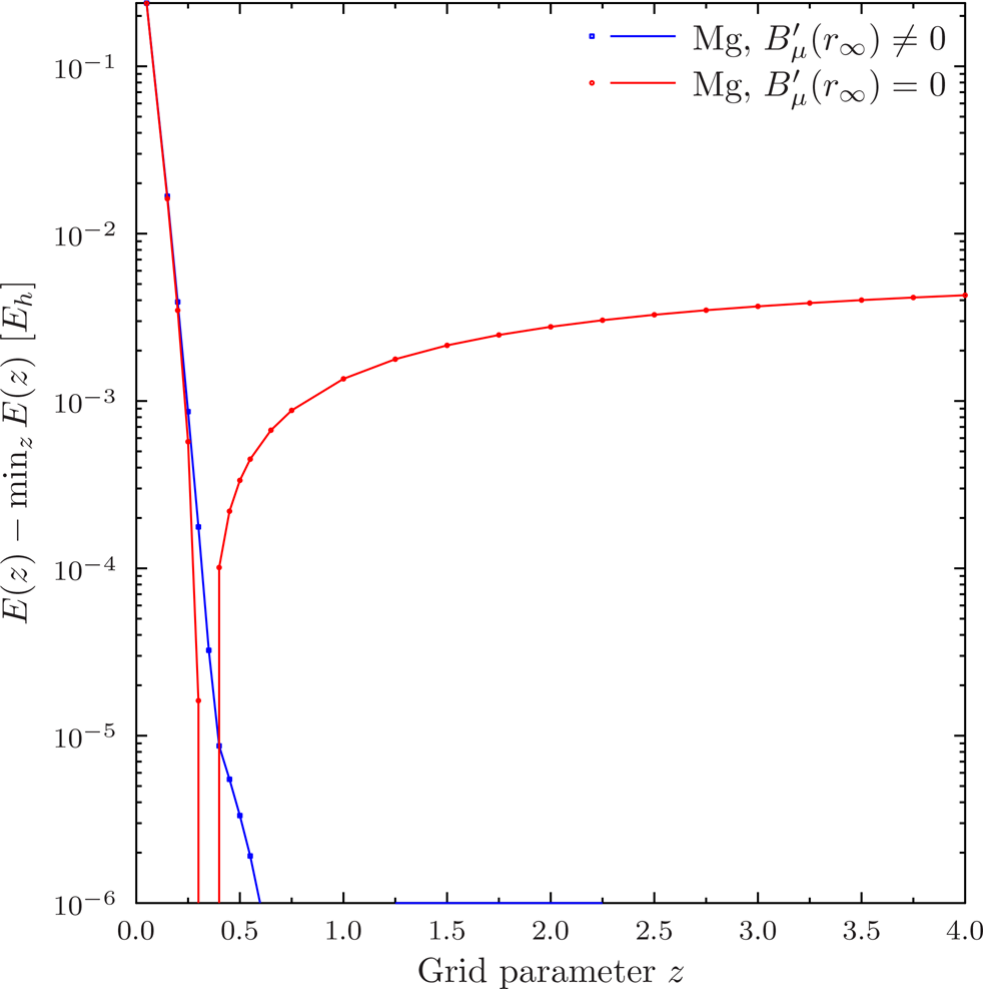
Dependence of the PBE total energy of Mg with *r*_∞_ = 4*a*_0_ on the grid parameter *z* in eq 3 with five 8-node HIP radial elements. Note logarithmic *y* axis.

In any case, convergence of *E*[*B*_μ_^′^(*r*_∞_) = 0] to the CBS limit is slow, and it makes sense to ask whether the grid could be improved. The element grid dependence is investigated in Figure 9. While the *B*_μ_^′^(*r*_∞_) ≠ 0 calculation shows negligible dependence on the value of the grid parameter *z*, as the calculations with *z* ≥ 0.6 are converged to the sub-*μE*_*h*_ accuracy, the *B*_μ_^′^(*r*_∞_) = 0 curve has a sharp minimum around *z* = 0.33, which is strikingly different from the recommended value *z* = 2.0 of ref (15). This indicates that the solution for *B*_μ_^′^(*r*_∞_) = 0 is apparently sensitive to the description of the wave function in the region near *r*_∞_, as such a choice places much more elements in the far-valence region than near the core, which furthermore suggests that CBS limit studies are impractical for the *B*_μ_^′^(*r*_∞_) = 0 boundary condition.

Combined with the realization that *E*[*B*_μ_^′^(*r*_∞_) ≠ 0] is clearly an upper bound for *E*[*B*_μ_^′^(*r*_∞_) = 0], since the latter calculation has a constraint missing in the former one, we are forced to conclude that the added control on the derivative offered by the HIP basis does not appear to offer further benefits over LIPs for building NAO basis sets.

Finally, because of the slow convergence seen in the *E*[*B*_μ_^′^(*r*_∞_) = 0], our advice is not to remove the function describing the derivative at *r*_∞_ as was done in section 3.2 to make the LIP and HIP calculations have exactly the same total number of radial basis functions, since keeping the extra function is hugely important for quick convergence in cases where *r*_∞_ is not converged to the free-atom limit.

## 4 Summary and Discussion

We have discussed how the numerical basis functions χ_μ_(*r*) = *B*_μ_(*r*)/*r* for atomic calculations can be evaluated in a numerically stable manner at points close to the origin. When a Taylor expansion of a lower polynomial order than that of *B*_μ_(*r*) is employed, the error made in the *d*th derivative at the switching point χ_μ_^(*d*)^(*R*) increases with the order of the derivative *d*. However, if the order of the Taylor expansion matches that of *B*_μ_(*r*), the Taylor series is accurate for all *d* and we recommend the use of such small-*r* expansions for all atomic calculations.

We have described the implementation of analytic first-order Hermite interpolating polynomials (HIPs) and numerical general-order Hermite interpolating polynomials, and studied their use in atomic electronic structure calculations. We have shown that HIPs can successfully be used in combination with a large number of nodes as well as with nonuniform finite element grids, where they afford results that are as good as or even better than those obtained with Lagrange interpolating polynomials (LIPs) used in our previous works.^15,17^ Furthermore, we demonstrated with the zinc atom that the grid dependence of LDA, GGA and meta-GGA functionals is similar to that of Hartree–Fock (HF) theory. (For a further application of 10-node HIPs, see ref (78) on the study of a recently proposed analytic, regularized nuclear Coulomb potential.)

We studied the importance of the HIP basis in meta-GGA calculations on the hydrogen atom. Calculations in small numerical basis sets were used to demonstrate that the LIP basis can yield a discontinuous local kinetic energy density τ at element boundaries, while the first-order HIP basis can reproduce kinks in τ already in calculations at the HF level; only the second-order (or higher) HIP basis is guaranteed to make τ smooth across element boundaries. We also identified self-consistent field (SCF) convergence problems with otherwise well-behaved τ-dependent meta-GGA functionals if a small numerical basis was employed, and explained this by the insufficient flexibility of such numerical basis sets. Finally, we showed that given a sufficiently large numerical basis set, all three choices for the shape functions reproduced similar results for HF and τ-dependent meta-GGA functionals for the hydrogen atom.

Our self-consistent calculations on hydrogen examined a large selection of functionals, including TPSS, TASKCC, r^2^SCAN, the whole Minnesota family—M05, M05-2X, M06, M06-2X, M06-SX, M06-L, revM06, revM06-L, M11, M11-L, revM11, MN12-SX, MN12-L, MN15, and MN15-L—and the three most recent Berkeley functionals ωB97X-V, B97M-V, and ωB97M-V without nonlocal correlation. TPSS, TASKCC, r^2^SCAN and all three Berkeley functionals were found to converge without problems in all three basis sets. M05, M05-2X, M06, M06-L, M06-2X, M08-SO, M11, and M11-L failed to either reach SCF convergence or the CBS limit despite the use of a large finite element basis set. The observed instabilities in these functionals are likely caused by oscillations and/or large values in the functionals’ enhancement factors.^61^ M08-HX was found to have converged close to the CBS limit, but it was found to exhibit nonphysical sharp features in ∇^2^*n*. Only the most recent Minnesota functionals—the 2020 M06-SX, the 2019 revM11, the 2018 revM06, the 2017 revM06-L, and the 2016 MN15 functional—as well as the older 2012 MN12-L and the 2012 MN12-SX functionals were found to converge without issues to the CBS limit. Interestingly, even though the aforementioned newer functionals share the same functional forms as the original ill-behaved parametrizations, the revised parameter values in M06-SX, revM11, and revM06-L appear to have removed the pathological behaviors in the earlier variants of the M06 and M11 type of functionals. Even though this part of the study was restricted to the hydrogen atom for simplicity, these results are directly useful also for calculations on other systems given that density functional approximations do not depend on the system. The numerical properties of Minnesota functionals and other recent meta-GGAs are studied on a wider variety of atoms in ref (79).

Although we have found that LIPs and HIPs work equally well for τ-dependent meta-GGAs when a large enough numerical basis set is used, calculations with density Laplacian dependent functionals may have more stringent requirements on the underlying shape function basis, as we exemplified with small HF calculations on the hydrogen atom. Higher-than-first order Hermite interpolating polynomials, which were studied numerically in this work, would be relevant for such efforts. General analytical formulas for such polynomials have been suggested in the literature,^80−82^ but they do not appear to have been used in practice. Further work should investigate the practical usefulness of the analytic expressions, as it is not clear whether implementations thereof will be sufficiently stable numerically.

## Supporting Information Available

Errors for approximate Taylor expansions from third to 16th order with a 15-node LIP basis and an 8-node analytical first-order HIP basis. Plots of τ and ∇^2^*n* for the hydrogen atom for all studied functionals (PDF)
