# Supplementary material for: Atomic Electronic Structure Calculations with Hermite Interpolating Polynomials
Source: J Phys Chem A. 2023 Apr 27;127(18):4180–93. doi: 10.1021/acs.jpca.3c00729 (PMC10184118; doi:10.1021/acs.jpca.3c00729)
Supplement: Supplementary file 1 — jp3c00729_si_001.pdf [file jp3c00729_si_001.pdf]

Supporting Information: Atomic Electronic Structure Calculations with Hermite Interpolating Polynomials

Susi Lehtola*,†,‡

†*Molecular Sciences Software Institute, Blacksburg, Virginia 24061, United States*
‡*Department of Chemistry, University of Helsinki, P.O. Box 55, FI-00014 University of Helsinki, Finland*

E-mail: susi.lehtola@alumni.helsinki.fi

The convergence of the Taylor expansion of the basis functions near the nucleus is shown in figure S1 for a 15-node LIP basis and figure S2 for an 8-node HIP basis, both with 5 radial elements. The case for 25 radial elements is shown in figures S3 and S4, respectively.

The plots for τ and $\nabla^2 n$ for the studied functionals from the calculations employing five radial elements and the high-order numerical basis sets are shown in

- figure S5 for the HF calculation
- figure S6 for the TPSS calculation
- figure S7 for the TASKCC calculation
- figure S8 for the M05 calculation
- figure S9 for the M06 calculation
- figure S10 for the M06-SX calculation
- figure S11 for the M06-L calculation
- figure S12 for the revM06 calculation
- figure S13 for the revM06-L calculation
- figure S14 for the M08-SO calculation
- figure S15 for the M08-HX calculation
- figure S16 for the M11 calculation
- figure S17 for the revM11 calculation
- figure S18 for the M11-L calculation
- figure S19 for the MN12-SX calculation
- figure S20 for the MN12-L calculation
- figure S21 for the MN15 calculation
- figure S22 for the MN15-L calculation
- figure S23 for the ωB97X-noV calculation
- figure S24 for the B97M-noV calculation
- figure S25 for the ωB97M-noV calculation
- figure S26 for the r^{2}SCAN calculation

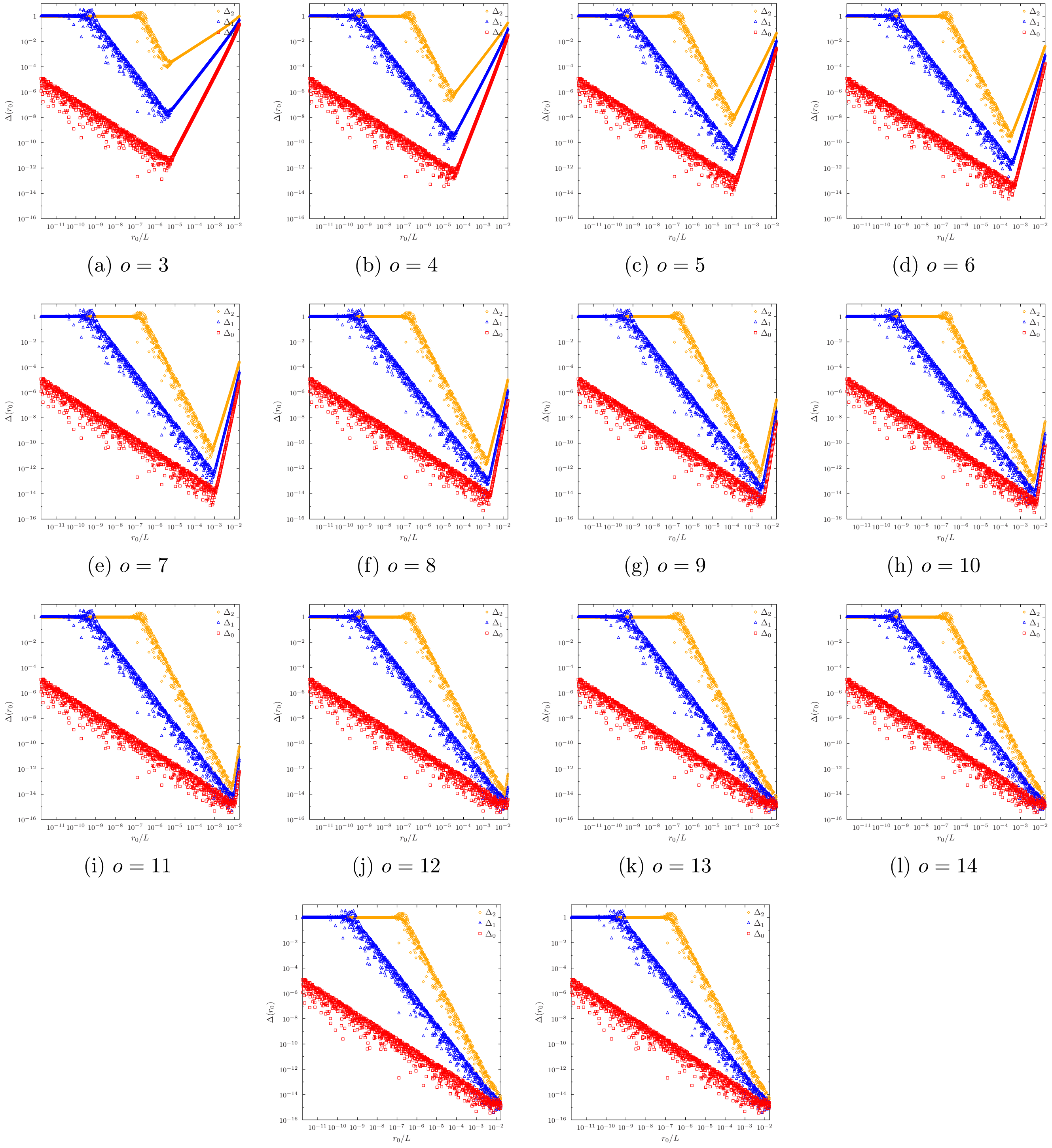

(a) $o = 3$ (b) $o = 4$ (c) $o = 5$ (d) $o = 6$

(e) $o = 7$ (f) $o = 8$ (g) $o = 9$ (h) $o = 10$

(i) $o = 11$ (j) $o = 12$ (k) $o = 13$ (l) $o = 14$

(m) $o = 15$ (n) $o = 16$

Figure S1: Error Δ in Taylor expansion as a function of the switching radius R for various orders o of the Taylor expansion, for a 15-node LIP basis with 5 elements; the first non-nuclear node is at $r_2/L \approx 0.017377$. Convergence is reached at $o = 14$, matching the polynomial order of the 15-node LIP basis.

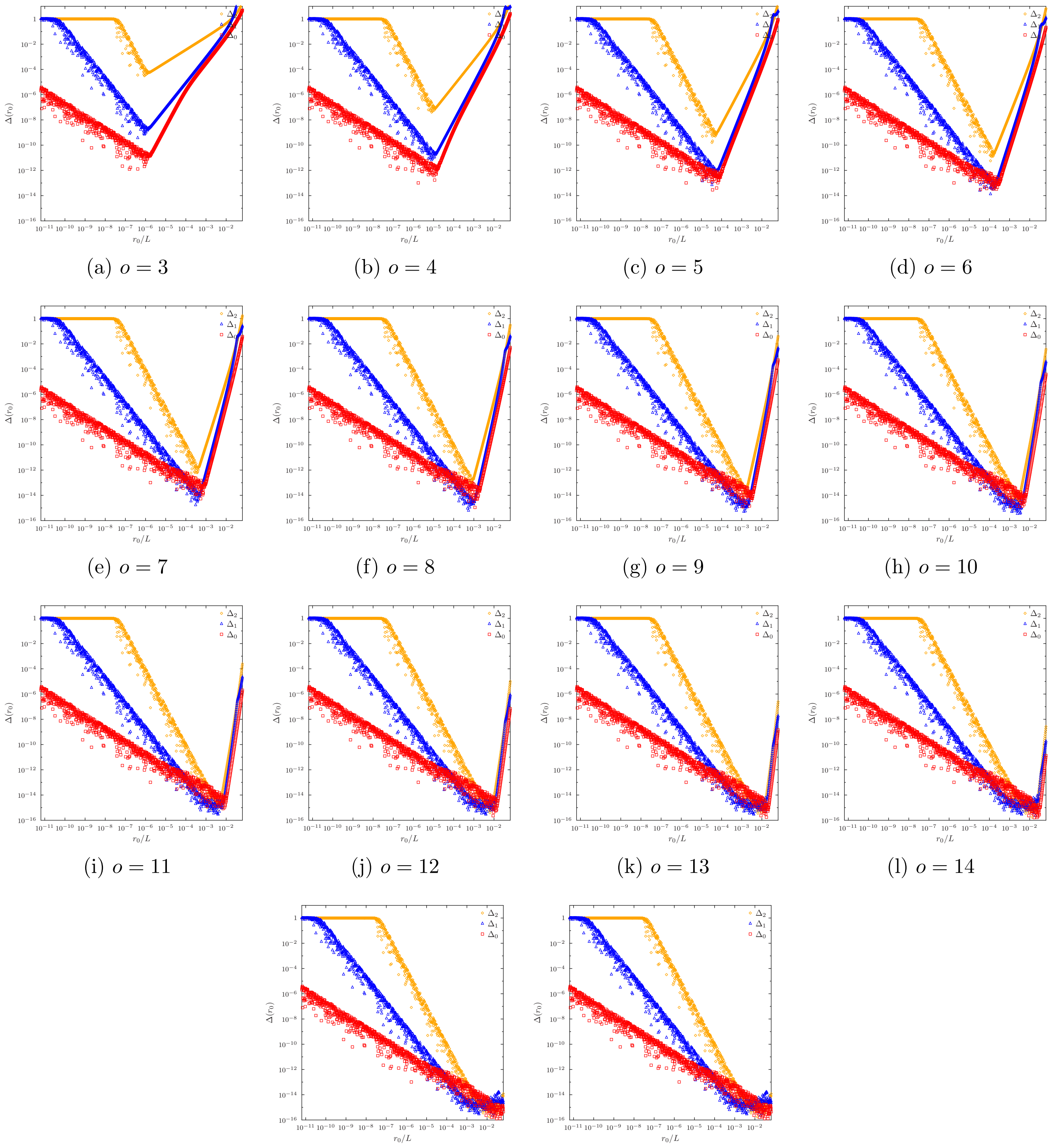

(a) $o = 3$ (b) $o = 4$ (c) $o = 5$ (d) $o = 6$

(e) $o = 7$ (f) $o = 8$ (g) $o = 9$ (h) $o = 10$

(i) $o = 11$ (j) $o = 12$ (k) $o = 13$ (l) $o = 14$

(m) $o = 15$ (n) $o = 16$

Figure S2: Error Δ in Taylor expansion as a function of the switching radius R for various orders o of the Taylor expansion, for a 8-node HIP basis with 5 elements; the first non-nuclear node is at $r_2/L \approx 0.064130$. Convergence is reached at $o = 15$, matching the polynomial order of the 8-node HIP basis.

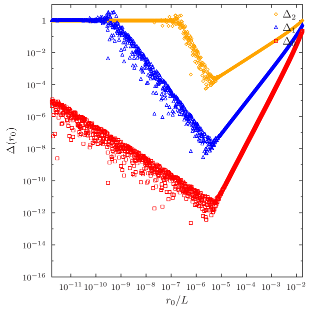

(a) $o = 3$

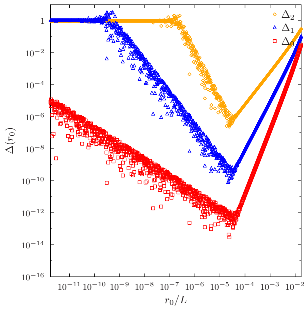

(b) $o = 4$

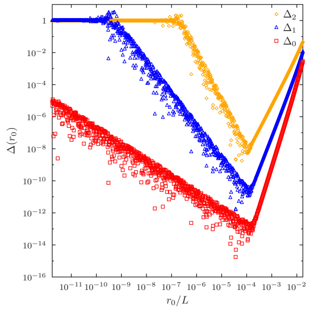

(c) $o = 5$

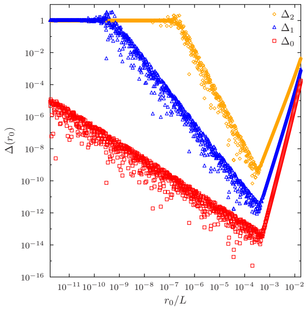

(d) $o = 6$

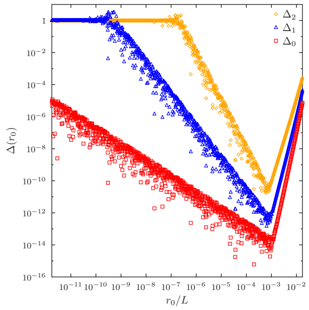

(e) $o = 7$

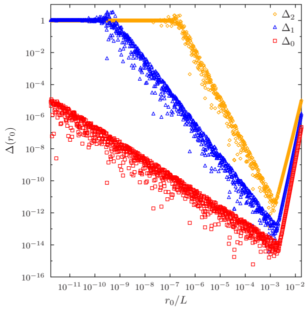

(f) $o = 8$

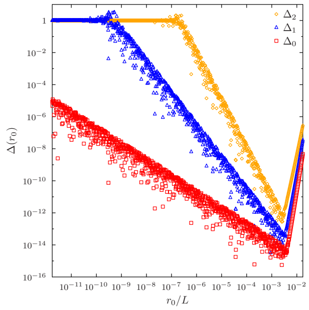

(g) $o = 9$

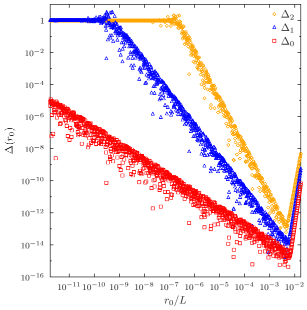

(h) $o = 10$

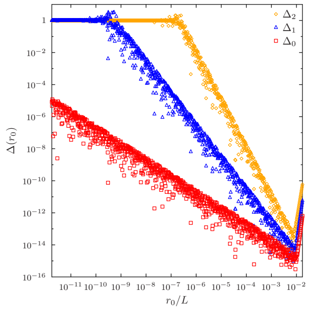

(i) $o = 11$

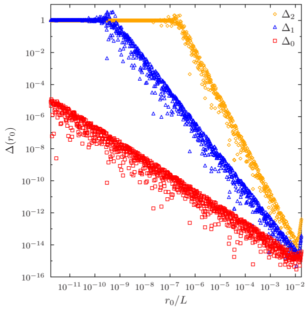

(j) $o = 12$

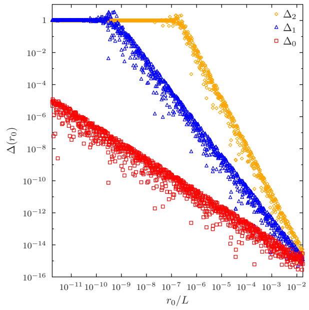

(k) $o = 13$

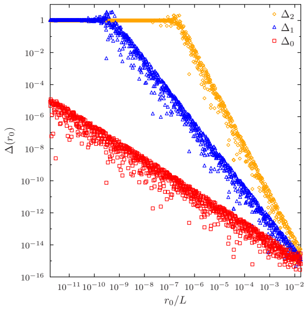

(l) $o = 14$

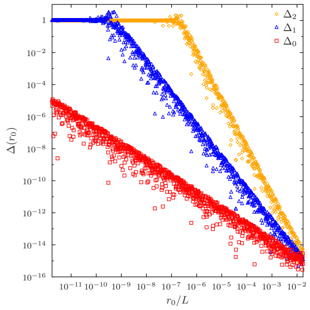

(m) $o = 15$

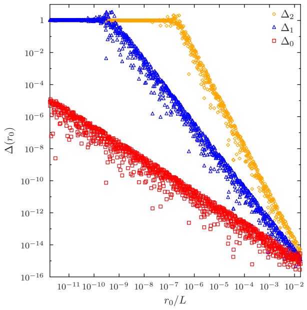

(n) $o = 16$

Figure S3: Error Δ in Taylor expansion as a function of the switching radius R for various orders o of the Taylor expansion, for a 15-node LIP basis with 5 elements; the first non-nuclear node is at $r_2/L \approx 0.017377$. Convergence is reached at $o = 14$, matching the polynomial order of the 15-node LIP basis.

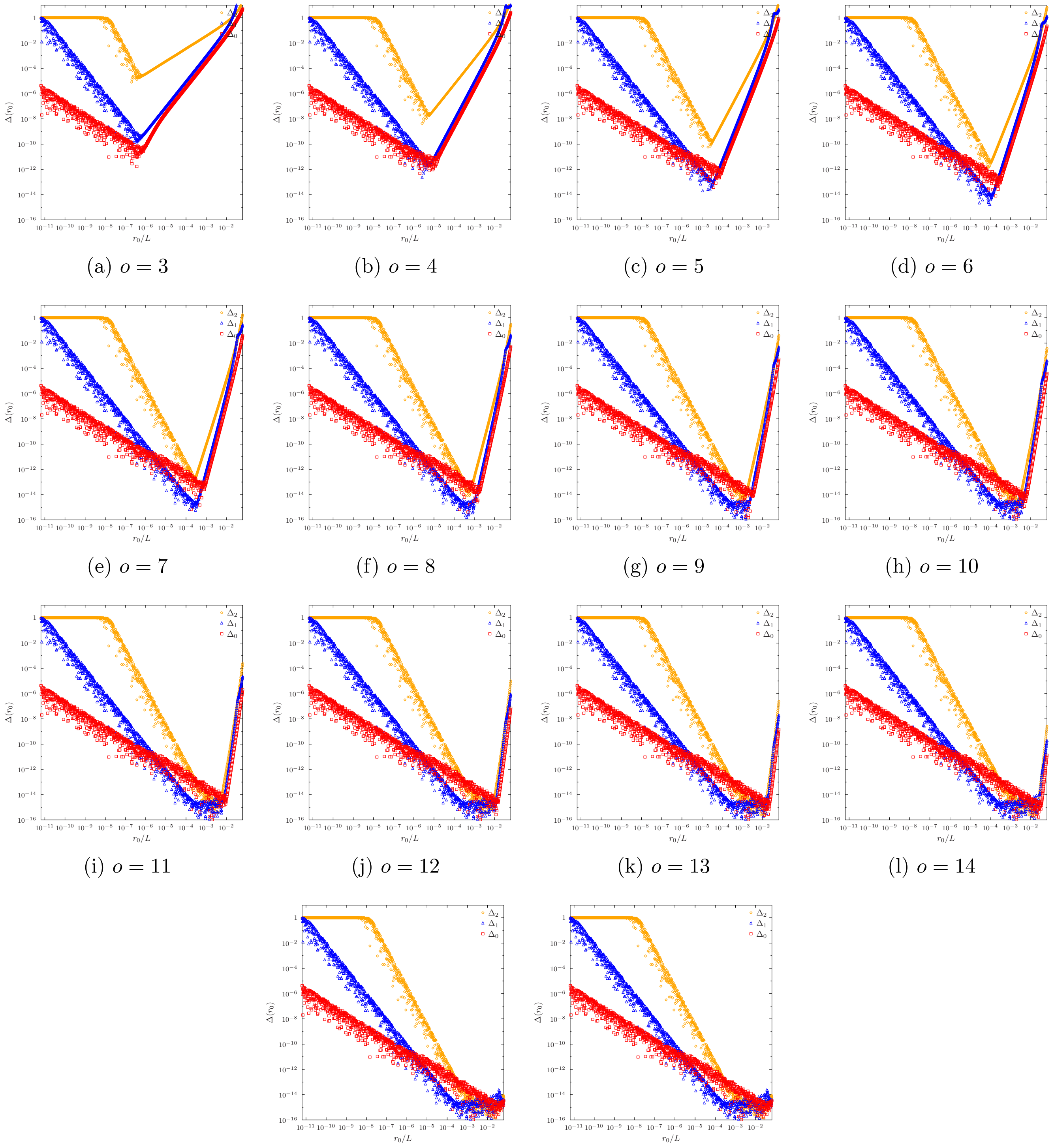

(a) $o = 3$ (b) $o = 4$ (c) $o = 5$ (d) $o = 6$

(e) $o = 7$ (f) $o = 8$ (g) $o = 9$ (h) $o = 10$

(i) $o = 11$ (j) $o = 12$ (k) $o = 13$ (l) $o = 14$

(m) $o = 15$ (n) $o = 16$

Figure S4: Error Δ in Taylor expansion as a function of the switching radius R for various orders o of the Taylor expansion, for a 8-node HIP basis with 5 elements; the first non-nuclear node is at $r_2/L \approx 0.064130$. Convergence is reached at $o = 15$, matching the polynomial order of the 8-node HIP basis.

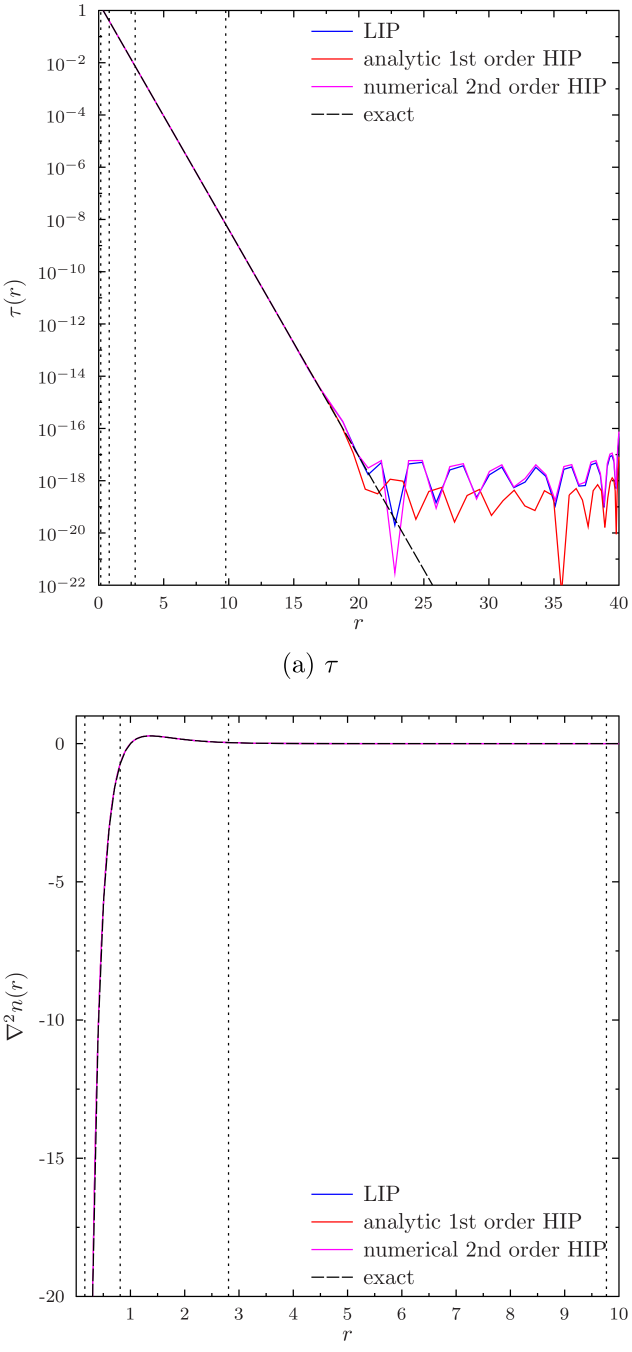

(a) τ

(b) $\nabla^2 n$

Figure S5: τ and $\nabla^2 n$ for the HF calculation.

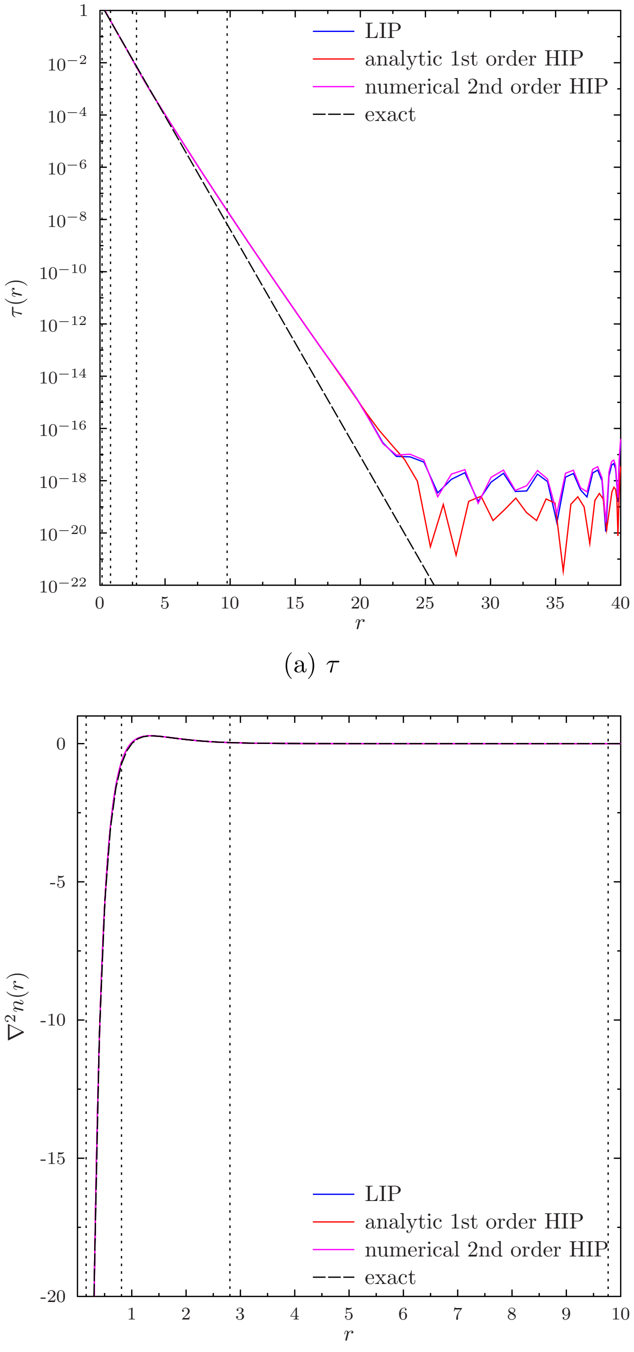

(a) τ

(b) $\nabla^2 n$

Figure S6: τ and $\nabla^2 n$ for the TPSS calculation.

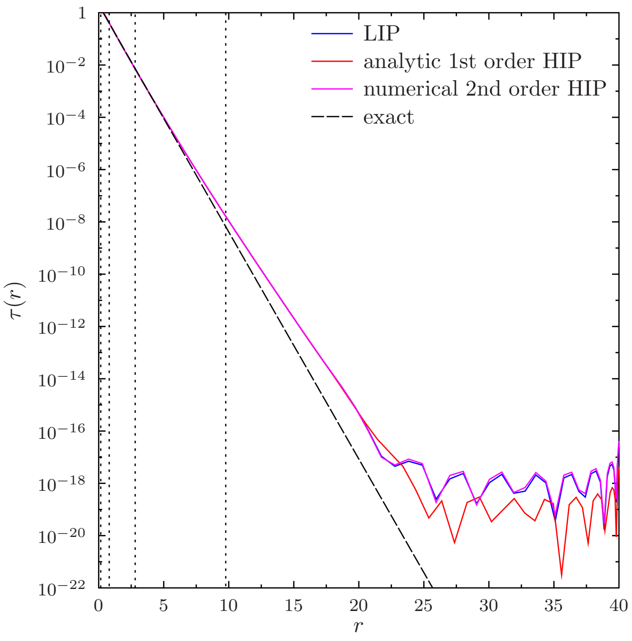

(a) τ

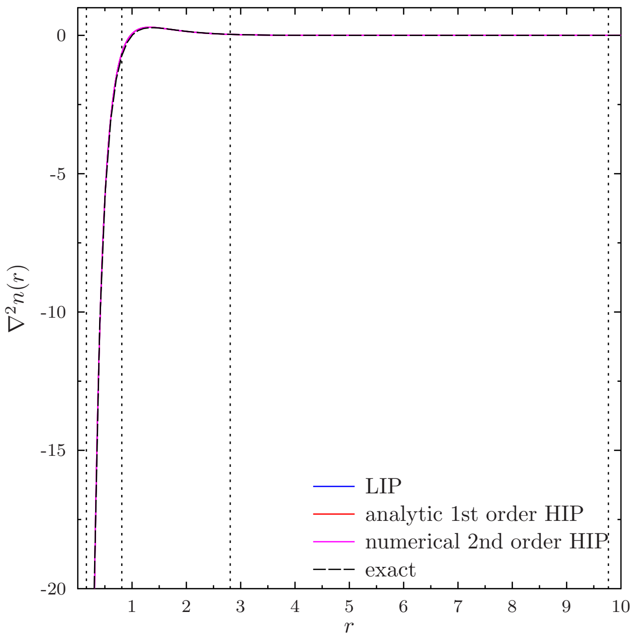

(b) $\nabla^2 n$

Figure S7: τ and $\nabla^2 n$ for the TASKCC calculation.

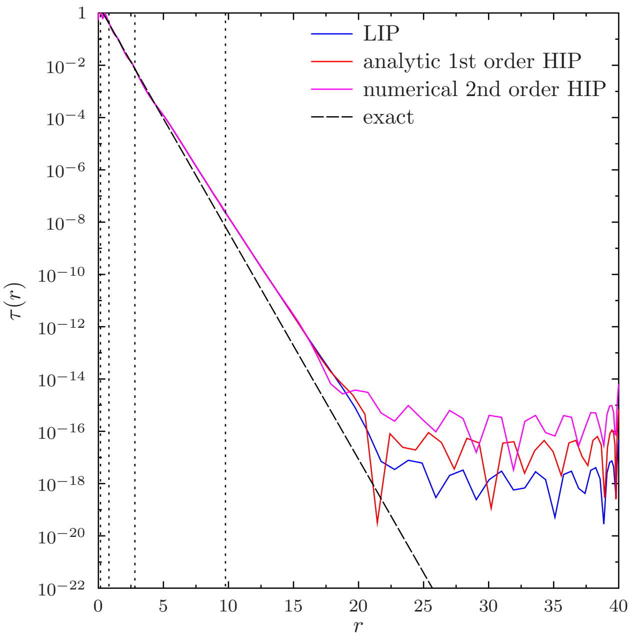

(a) τ

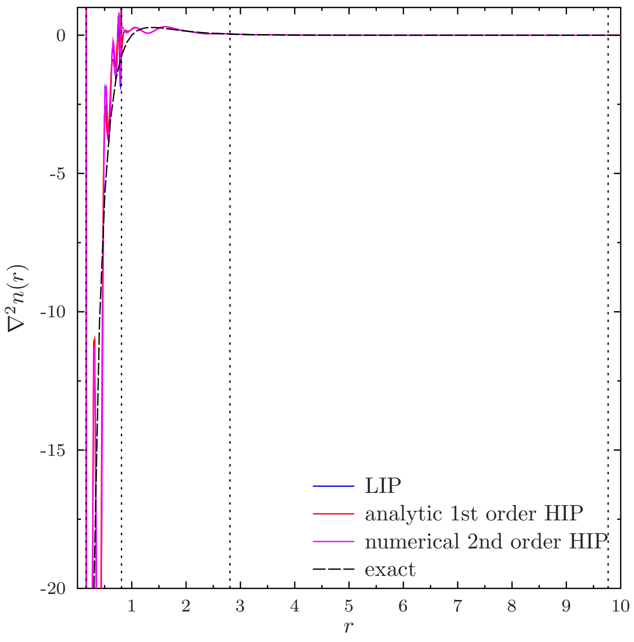

(b) $\nabla^2 n$

Figure S8: τ and $\nabla^2 n$ for the M05 calculation.

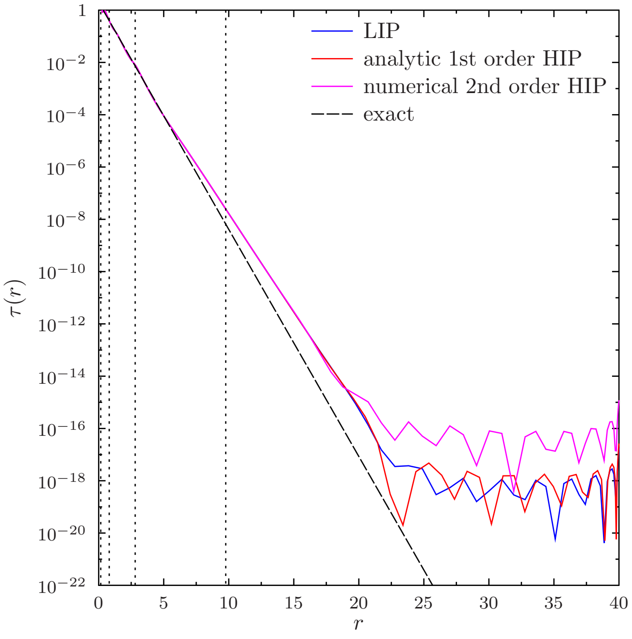

(a) τ

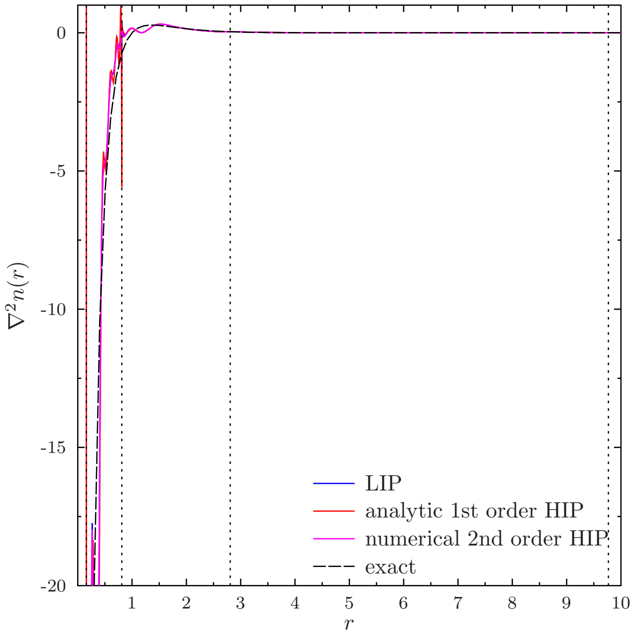

(b) $\nabla^2 n$

Figure S9: τ and $\nabla^2 n$ for the M06 calculation.

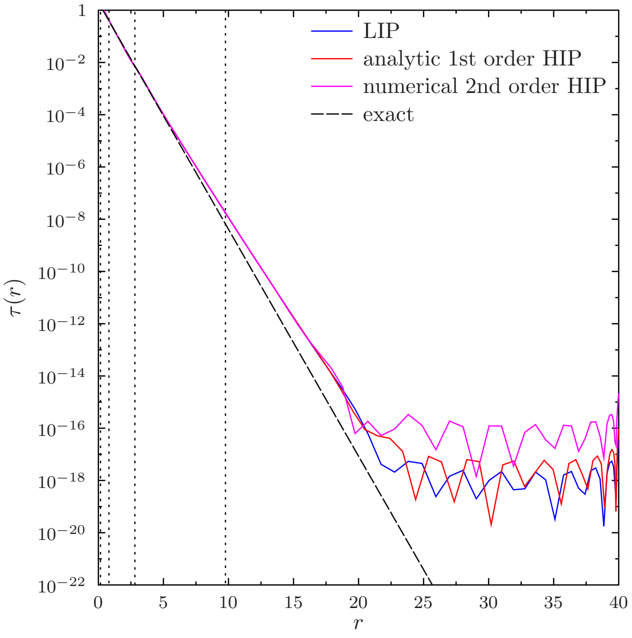

(a) τ

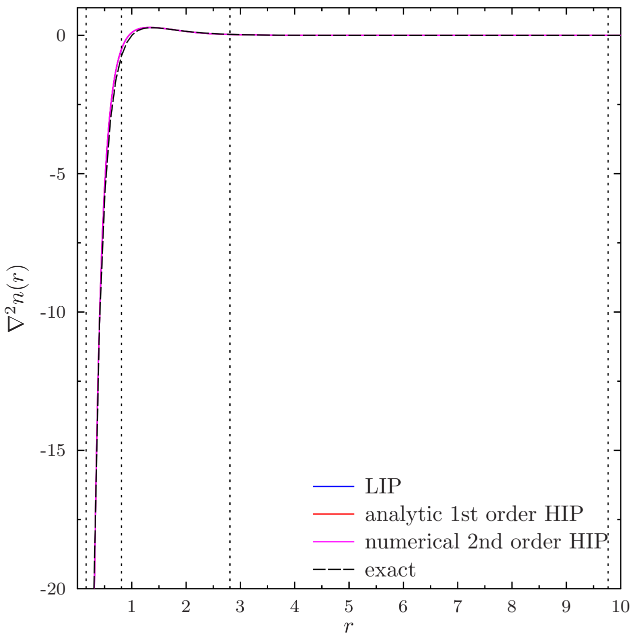

(b) $\nabla^2 n$

Figure S10: τ and $\nabla^2 n$ for the M06-SX calculation.

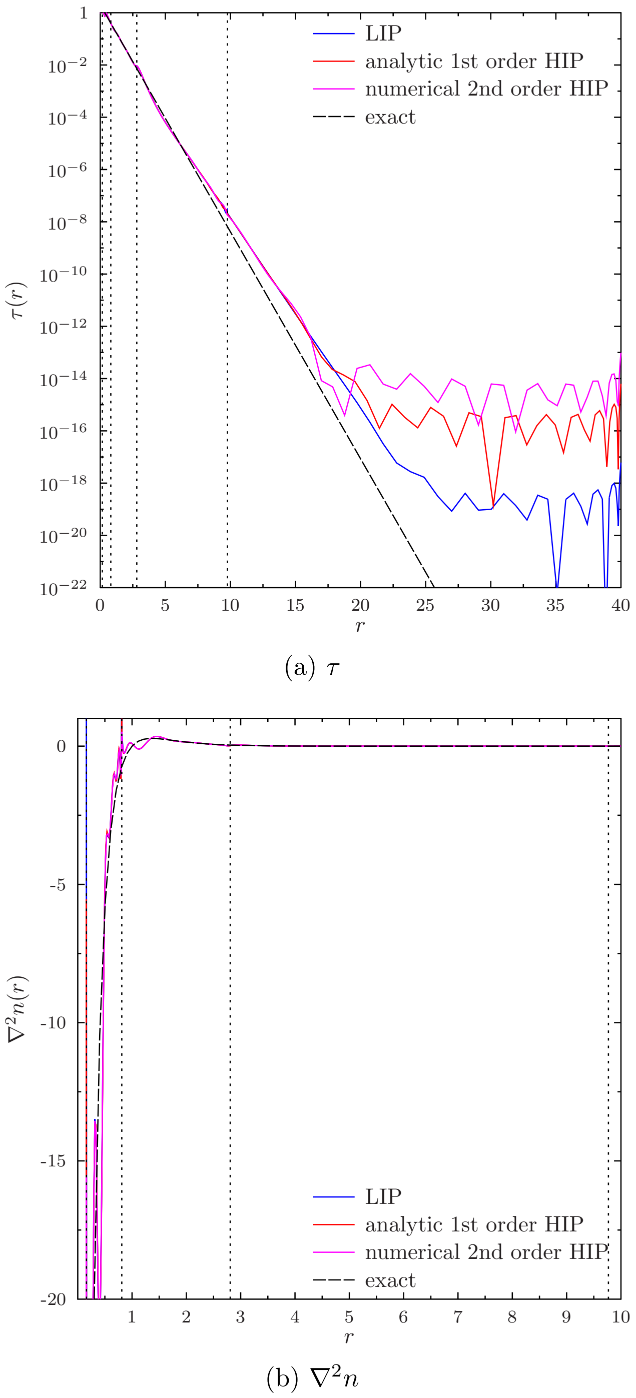

(a) τ

(b) $\nabla^2 n$

Figure S11: τ and $\nabla^2 n$ for the M06-L calculation.

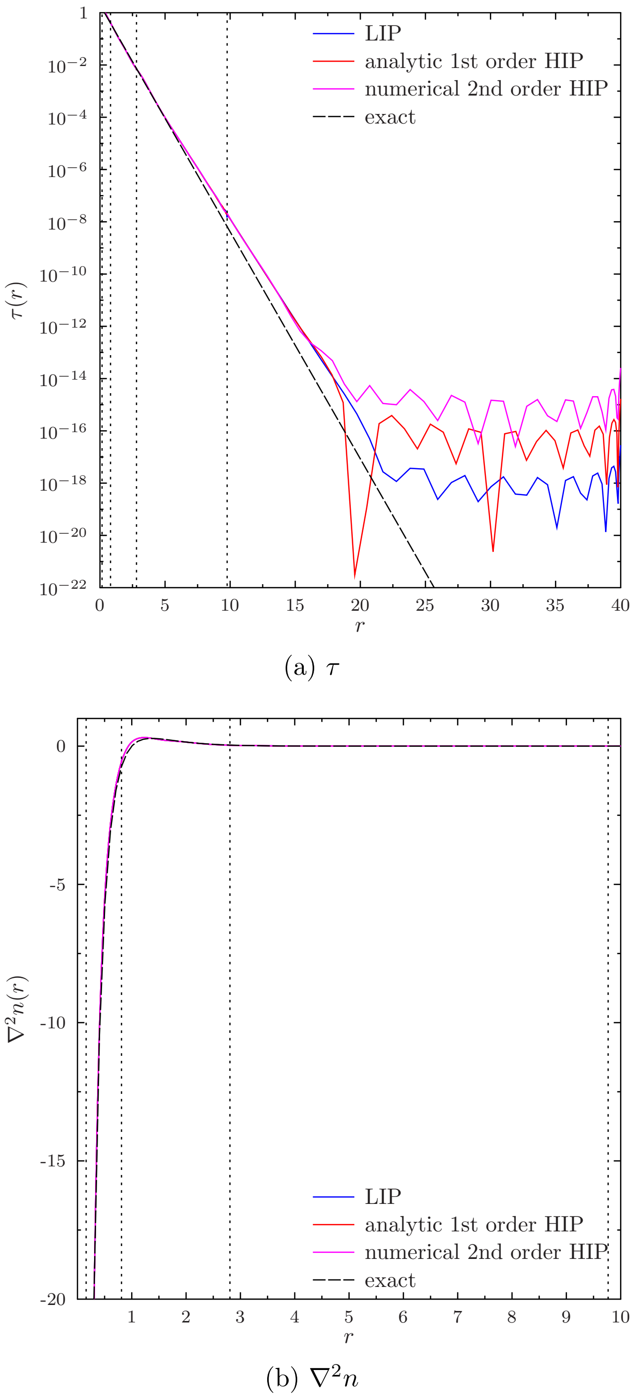

(a) τ

(b) $\nabla^2 n$

Figure S12: τ and $\nabla^2 n$ for the revM06 calculation.

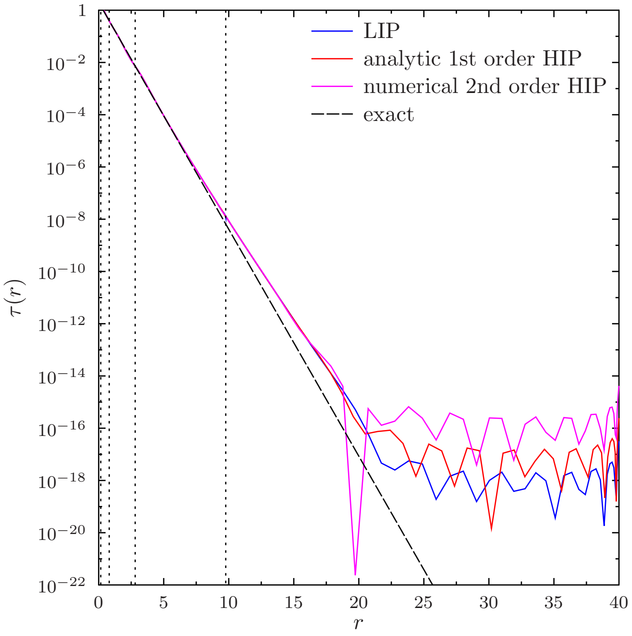

(a) τ

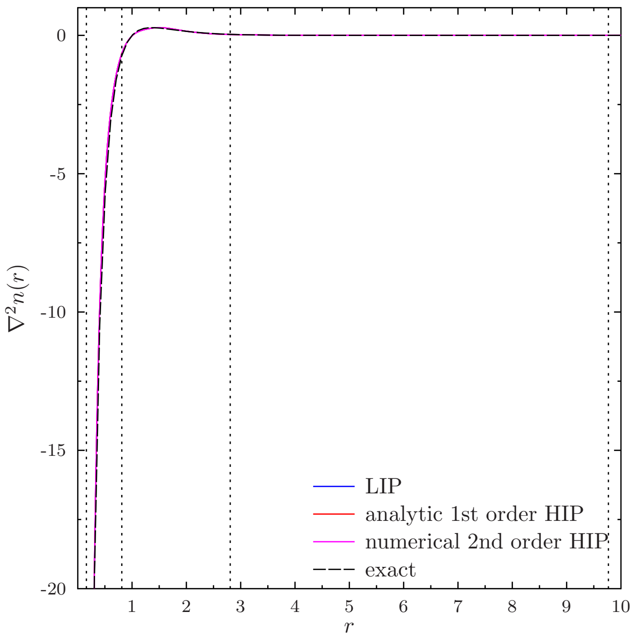

(b) $\nabla^2 n$

Figure S13: τ and $\nabla^2 n$ for the revM06-L calculation.

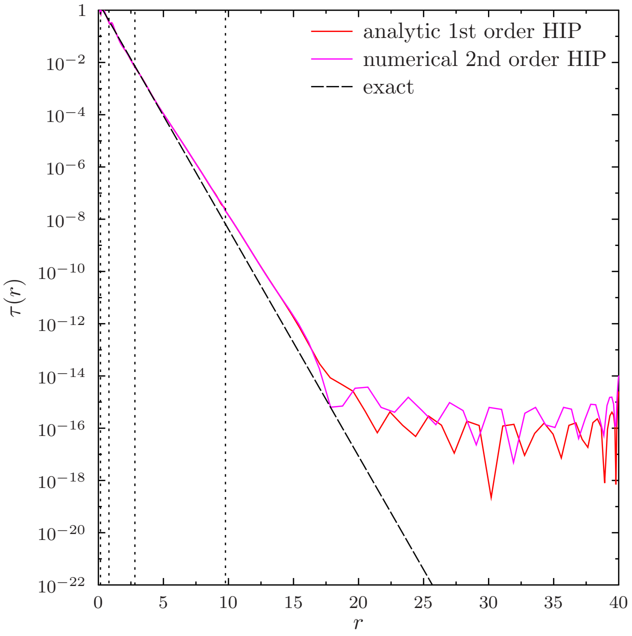

(a) τ

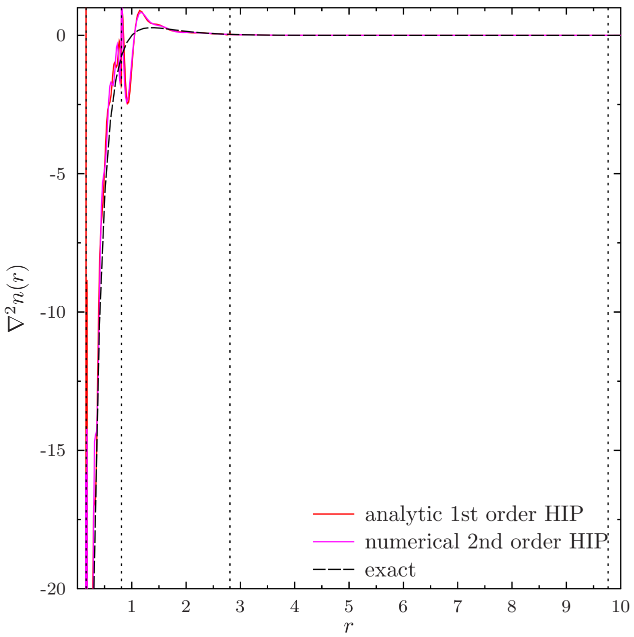

(b) $\nabla^2 n$

Figure S14: τ and $\nabla^2 n$ for the M08-SO calculation.

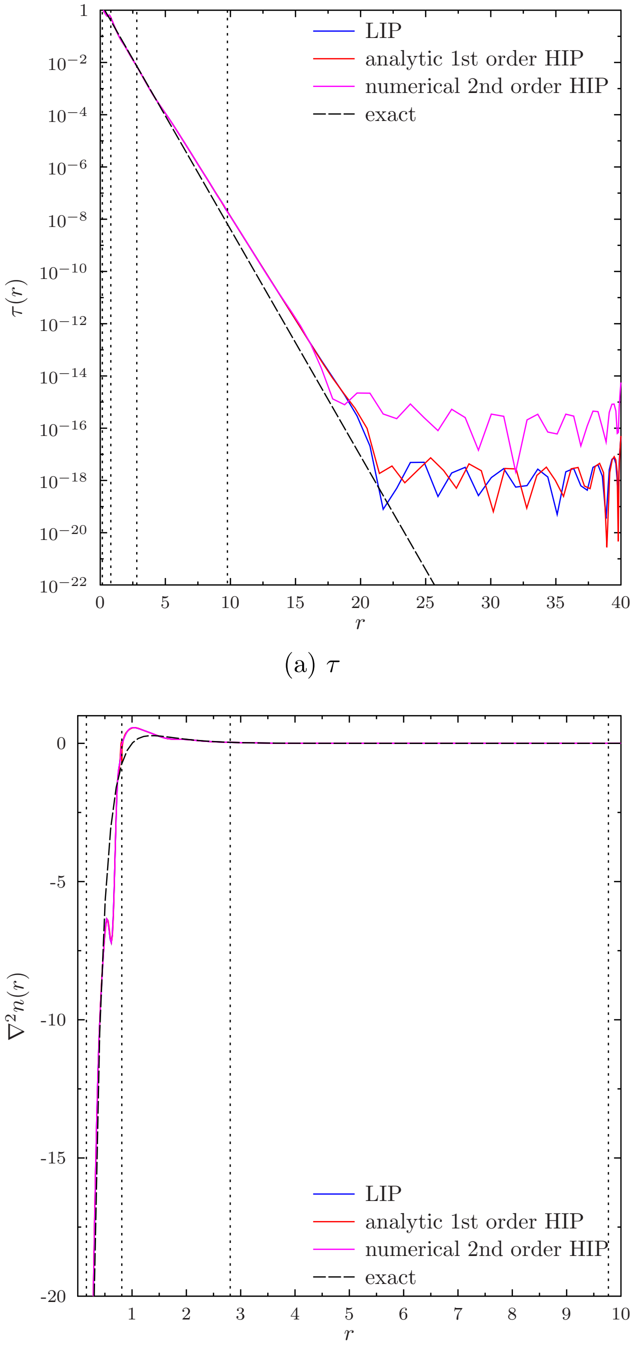

(a) τ

(b) $\nabla^2 n$

Figure S15: τ and $\nabla^2 n$ for the M08-HX calculation.

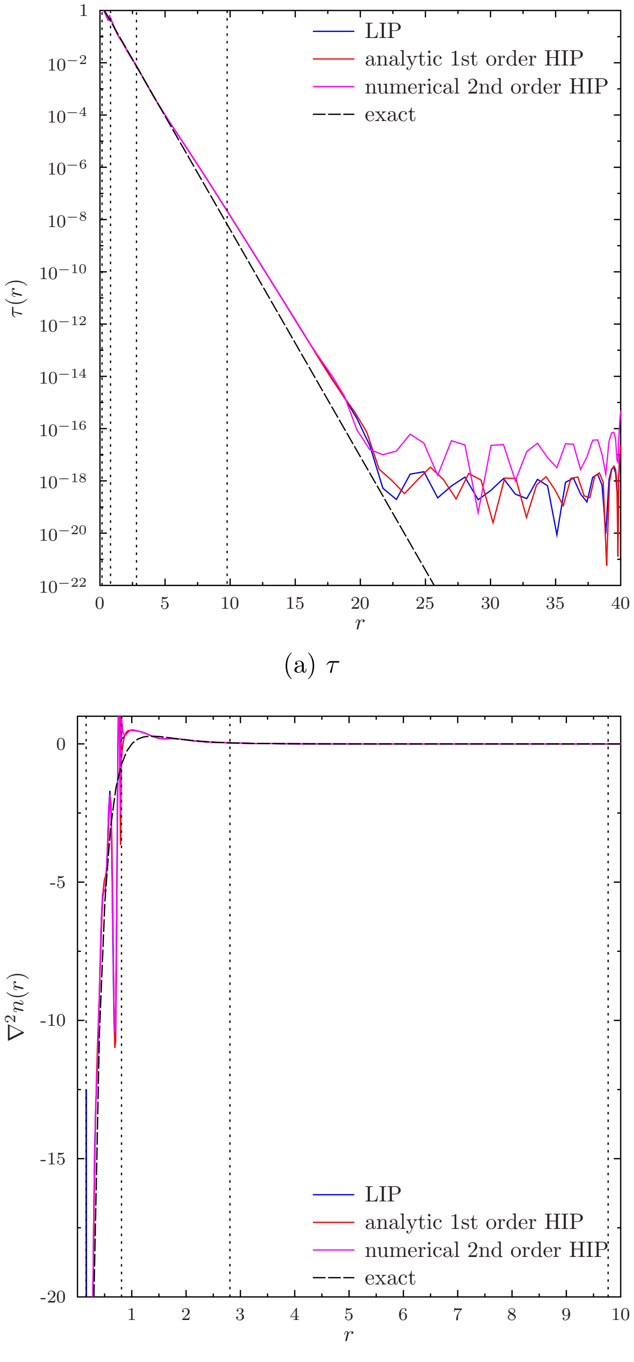

(a) τ

(b) $\nabla^2 n$

Figure S16: τ and $\nabla^2 n$ for the M11 calculation.

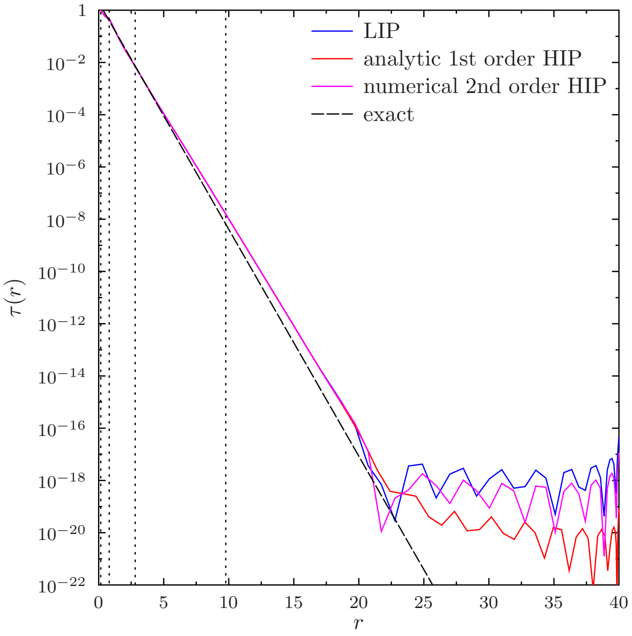

(a) τ

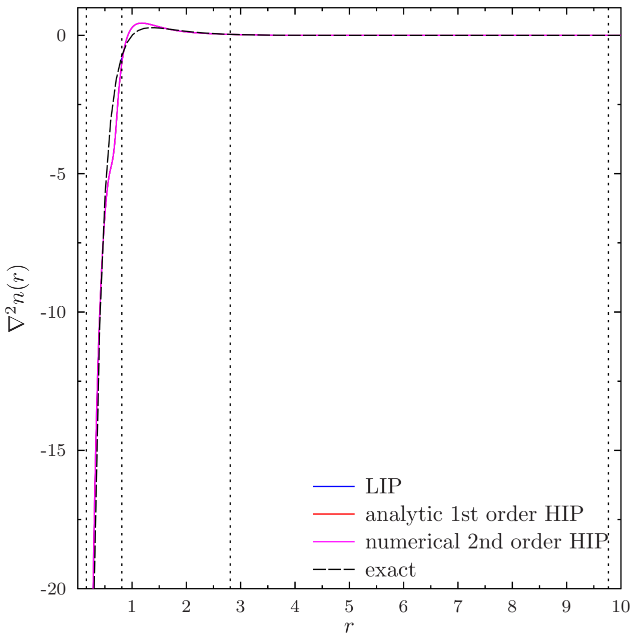

(b) $\nabla^2 n$

Figure S17: τ and $\nabla^2 n$ for the revM11 calculation.

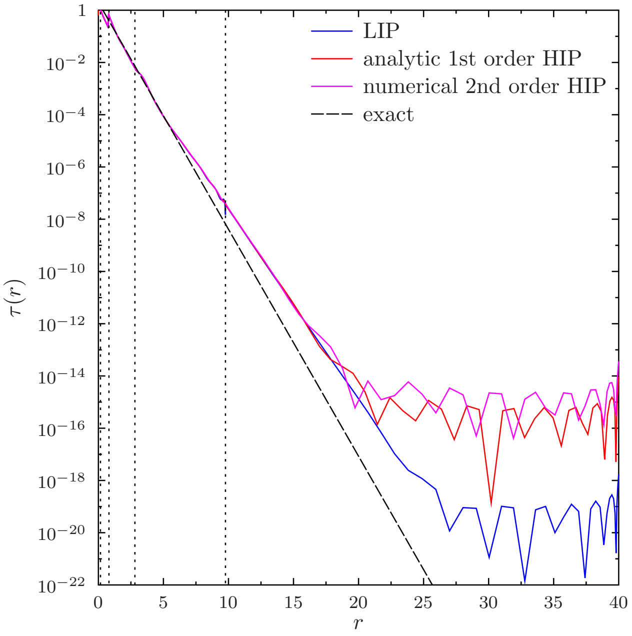

(a) τ

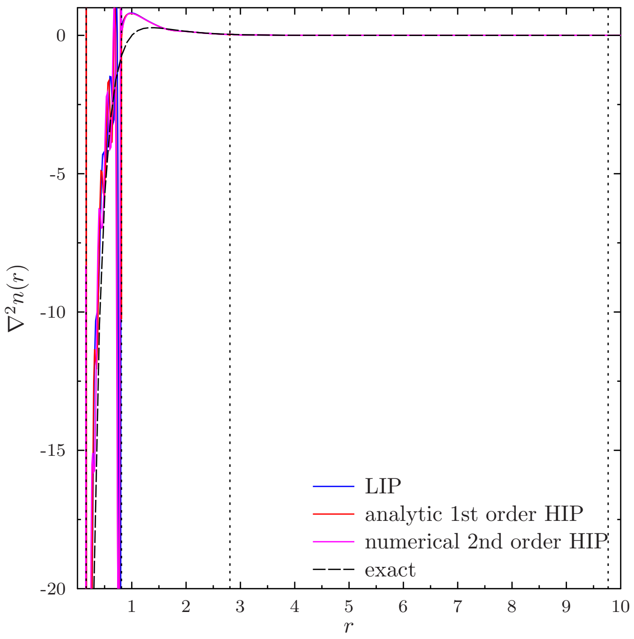

(b) $\nabla^2 n$

Figure S18: τ and $\nabla^2 n$ for the M11-L calculation.

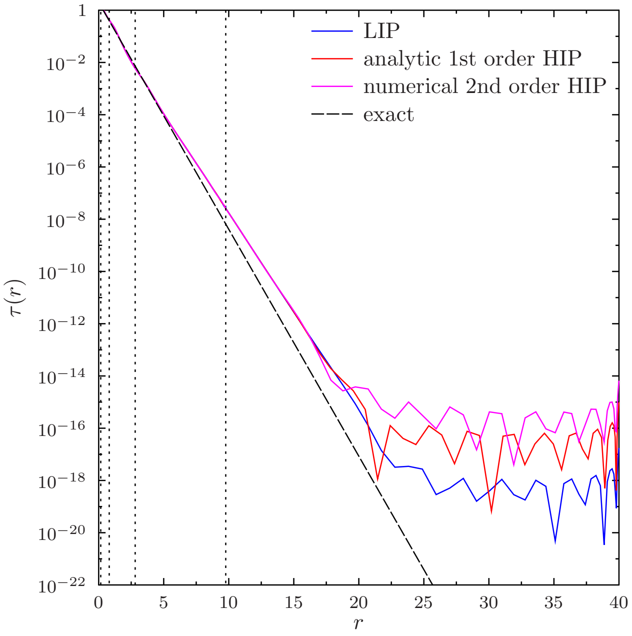

(a) τ

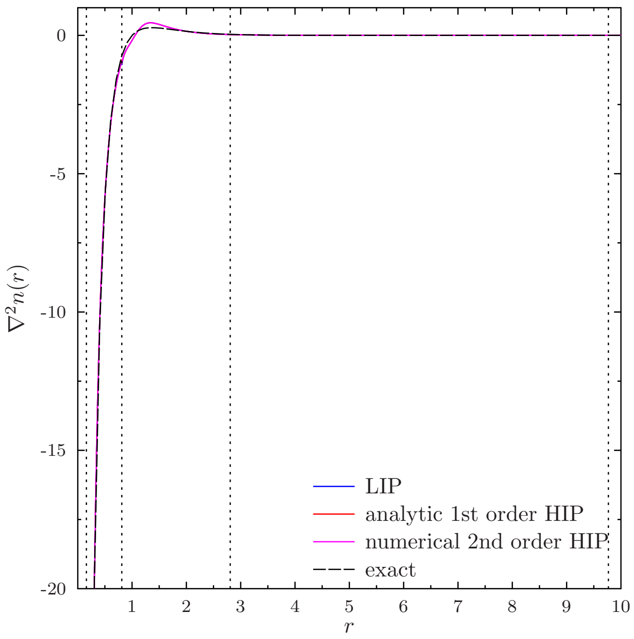

(b) $\nabla^2 n$

Figure S19: τ and $\nabla^2 n$ for the MN12-SX calculation.

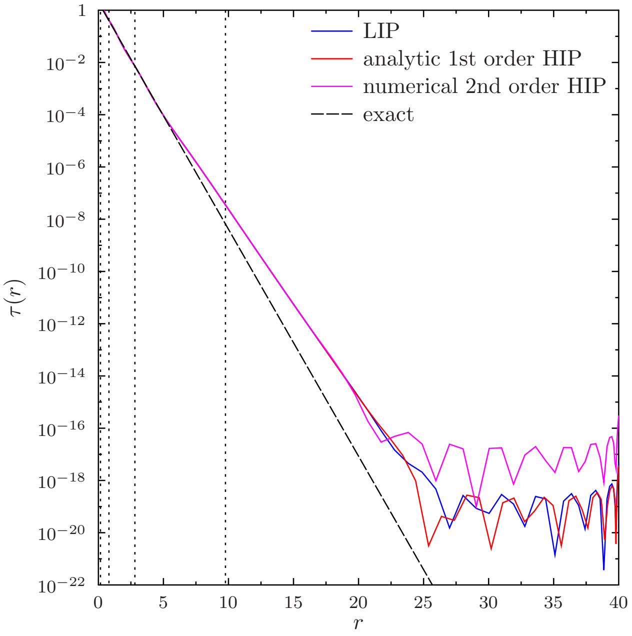

(a) τ

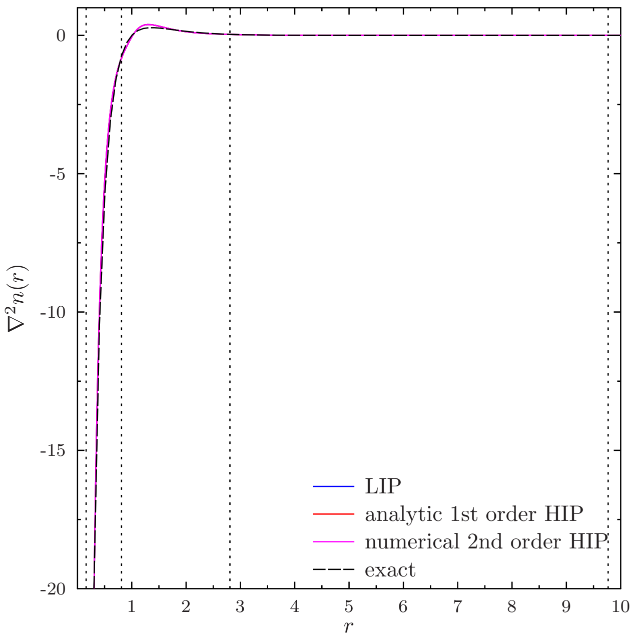

(b) $\nabla^2 n$

Figure S20: τ and $\nabla^2 n$ for the MN12-L calculation.

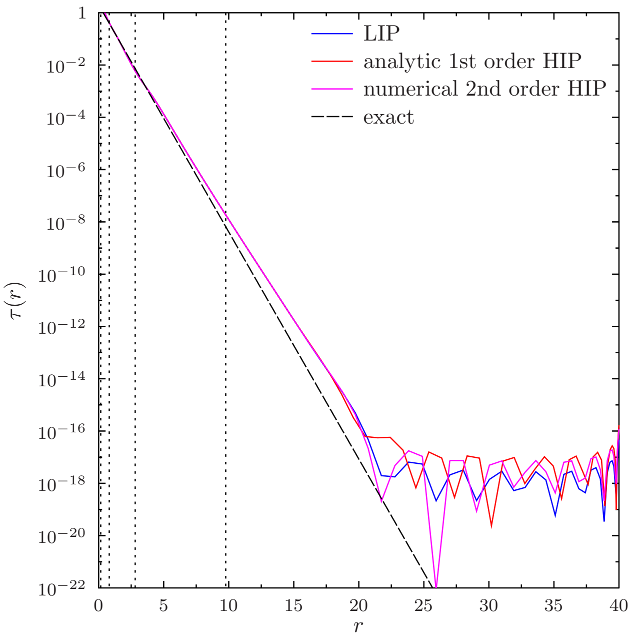

(a) τ

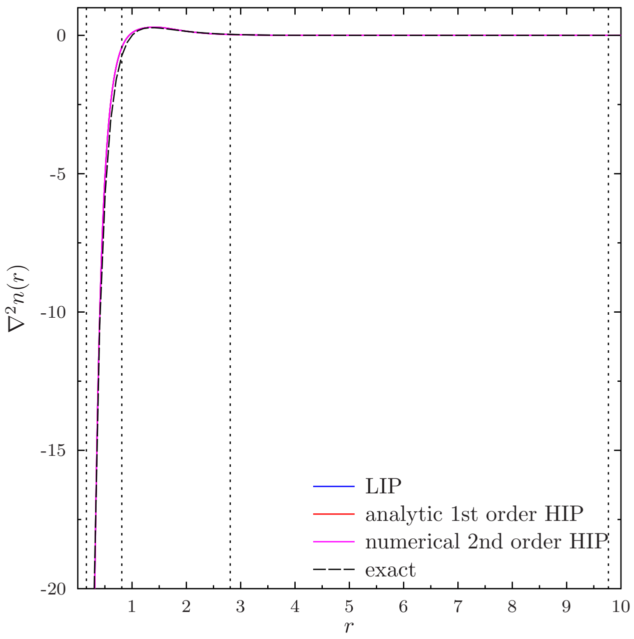

(b) $\nabla^2 n$

Figure S21: τ and $\nabla^2 n$ for the MN15 calculation.

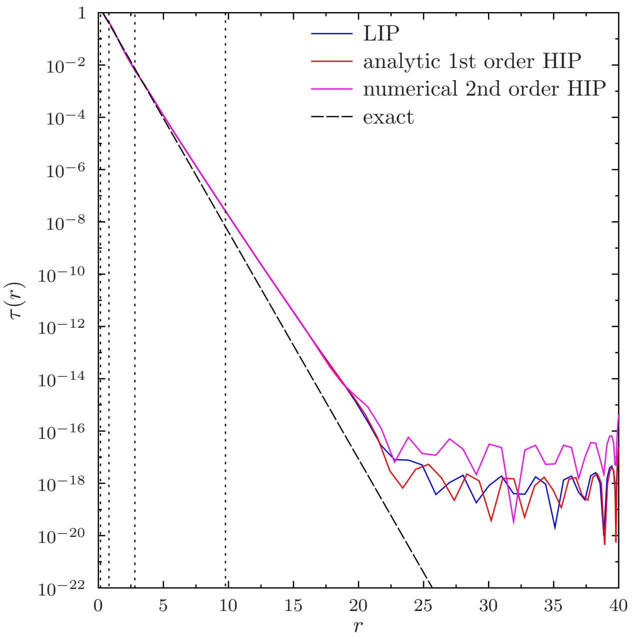

(a) τ

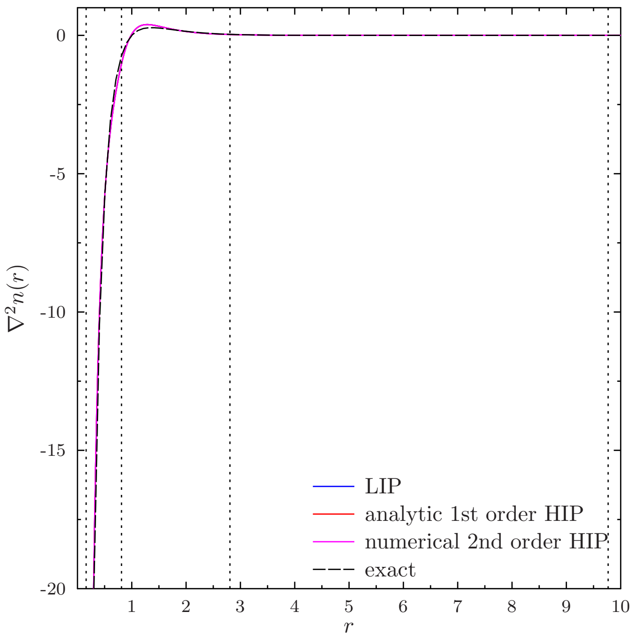

(b) $\nabla^2 n$

Figure S22: τ and $\nabla^2 n$ for the MN15-L calculation.

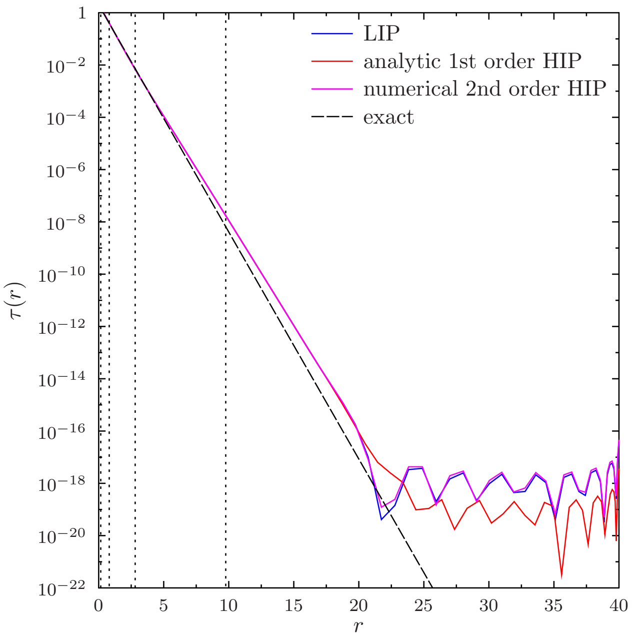

(a) τ

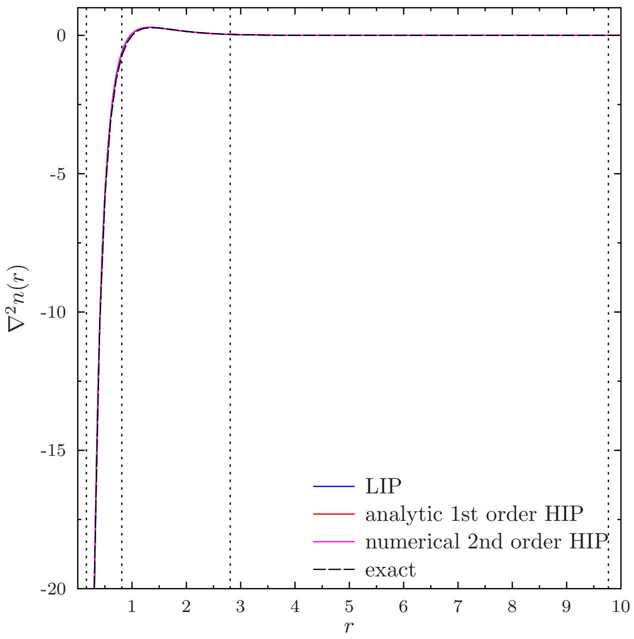

(b) $\nabla^2 n$

Figure S23: τ and $\nabla^2 n$ for the ωB97X-noV calculation.

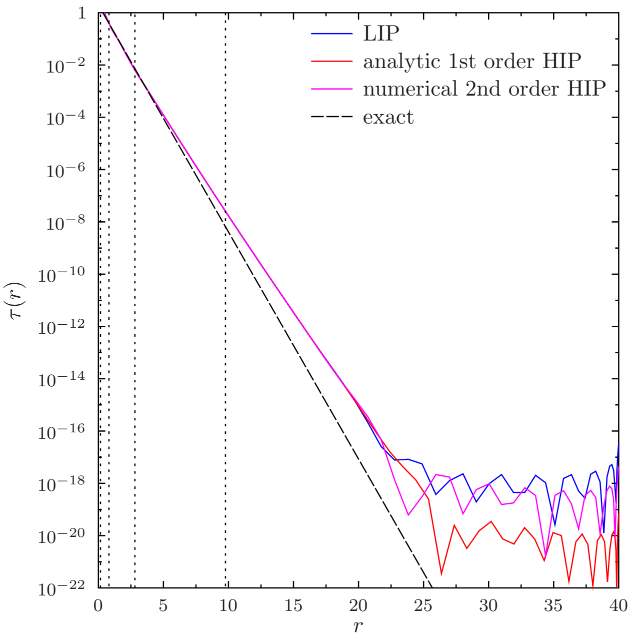

(a) τ

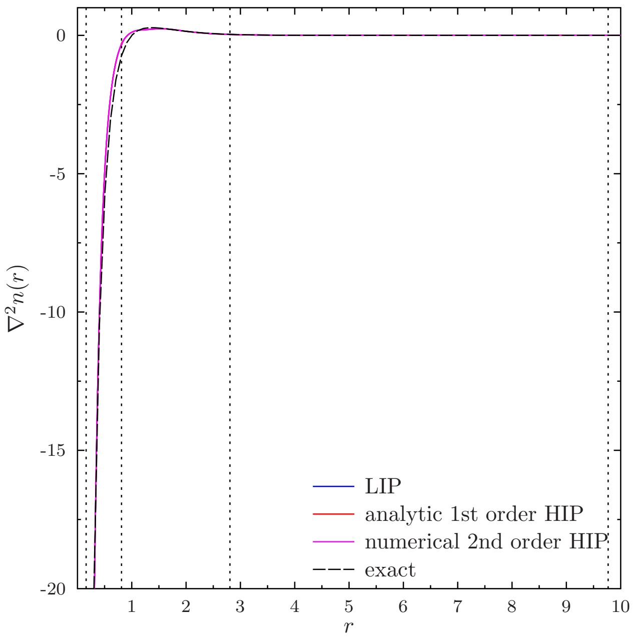

(b) $\nabla^2 n$

Figure S24: τ and $\nabla^2 n$ for the B97M-noV calculation.

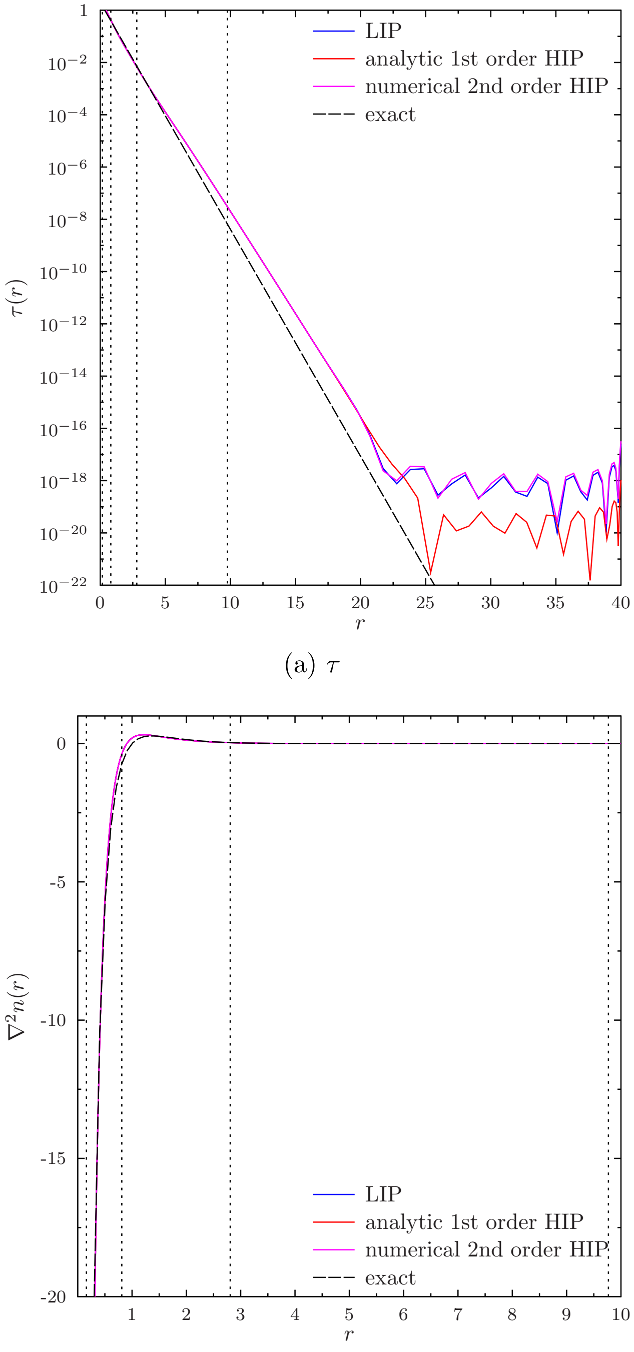

(a) τ

(b) $\nabla^2 n$

Figure S25: τ and $\nabla^2 n$ for the ωB97M-noV calculation.

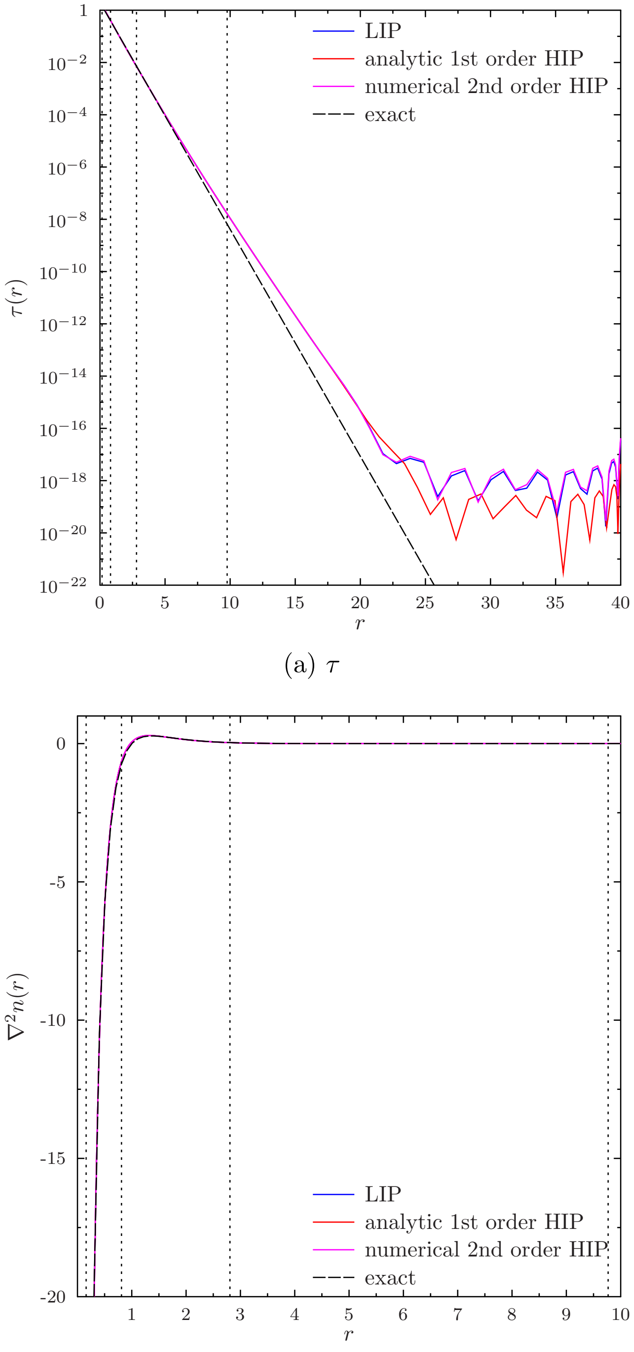

(a) τ

(b) $\nabla^2 n$

Figure S26: τ and $\nabla^2 n$ for the r^{2}SCAN calculation.
